# Prominosomes - a particular class of extracellular vesicles containing prominin-1/CD133?

**DOI:** 10.1186/s12951-025-03102-w

**Published:** 2025-01-29

**Authors:** Jana Karbanová, Kristina Thamm, Christine A. Fargeas, Ilker A. Deniz, Aurelio Lorico, Denis Corbeil

**Affiliations:** 1 Biotechnology Center (BIOTEC) and Center for Molecular and Cellular Bioengineering, Technische Universität Dresden, Tatzberg 47-49, 01307 Dresden, Germany; 2 Tissue Engineering Laboratories, Medizinische Fakultät der Technischen Universität Dresden, Fetscherstr. 74, 01307 Dresden, Germany; 3 College of Osteopathic Medicine, Touro University Nevada, 874 American Pacific Drive, Henderson, NV 89014 USA; 4 Tissue Engineering Laboratories, Biotechnology Center, Technische Universität Dresden, Tatzberg 47-49, 01307 Dresden, Germany; 5 Present Address: denovoMATRIX GmbH, Tatzberg 47, 01307 Dresden, Germany

**Keywords:** CD133, Cell signaling, Cilium, Ectosome, Exosome, Intercellular communication, Lipid droplet, Midbody, Microvillus, Stem cell

## Abstract

Extracellular membrane vesicles (EVs) offer promising values in various medical fields, e.g., as biomarkers in liquid biopsies or as native (or bioengineered) biological nanocarriers in tissue engineering, regenerative medicine and cancer therapy. Based on their cellular origin EVs can vary considerably in composition and diameter. Cell biological studies on mammalian prominin-1, a cholesterol-binding membrane glycoprotein, have helped to reveal new donor membranes as sources of EVs. For instance, small EVs can originate from microvilli and primary cilia, while large EVs might be produced by transient structures such as retracting cellular extremities of cancer cells during the mitotic rounding process, and the midbody at the end of cytokinesis. Here, we will highlight the various subcellular origins of prominin-1^+^ EVs, also called prominosomes, and the potential mechanism(s) regulating their formation. We will further discuss the molecular and cellular characteristics of prominin-1, notably those that have a direct effect on the release of prominin-1^+^ EVs, a process that might be directly implicated in donor cell reprogramming of stem and cancer stem cells. Prominin-1^+^ EVs also mediate intercellular communication during embryonic development and adult homeostasis in healthy individuals, while disseminating biological information during diseases.

**Graphical abstract:**

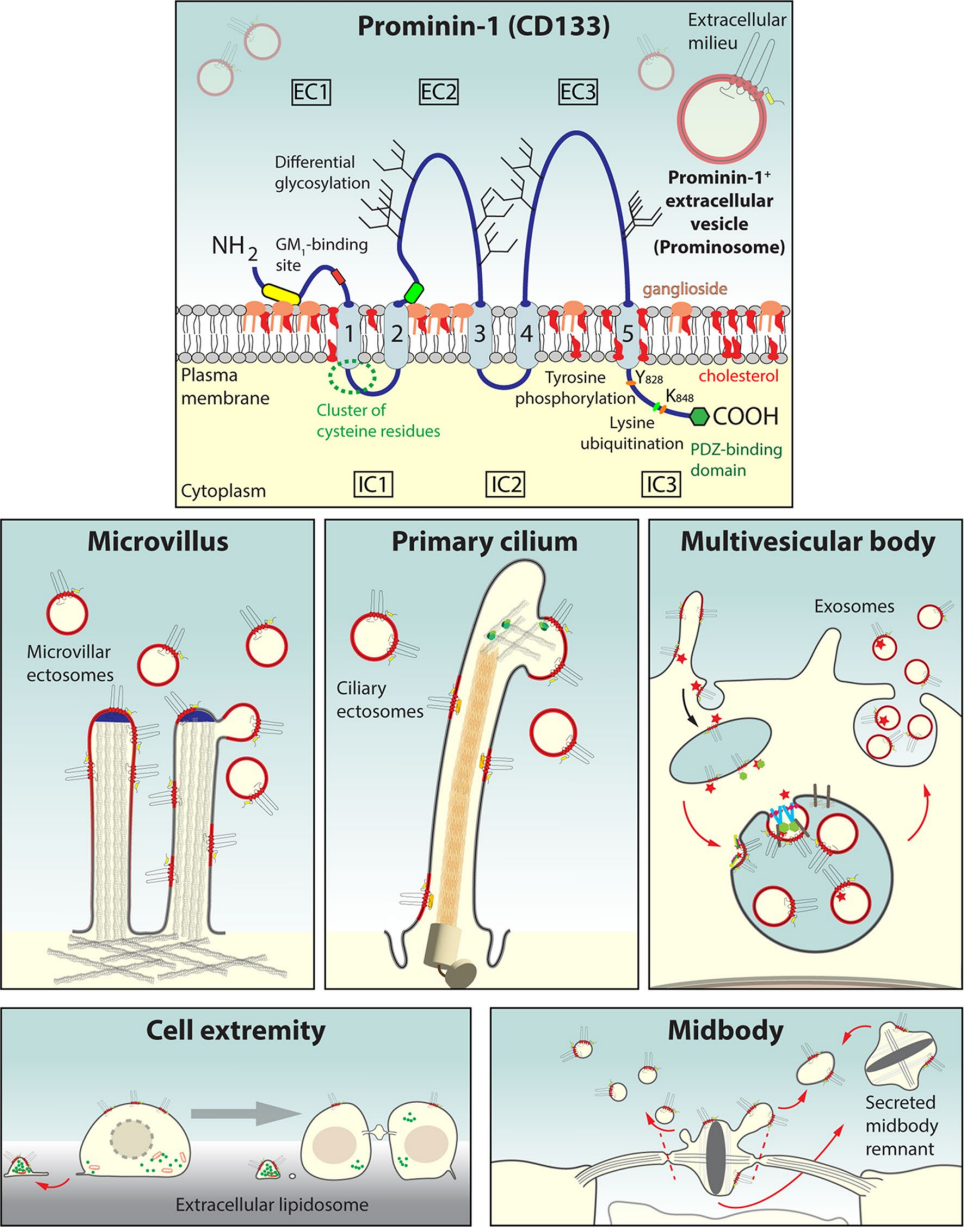

**Supplementary Information:**

The online version contains supplementary material available at 10.1186/s12951-025-03102-w.

## Introduction

Nowadays, the interest in extracellular membrane vesicles (EVs) is growing exponentially due to their role in many physiological processes and their potential therapeutical use for various medical applications [1]. Distinct nano- and micro-sized EVs have been described in the literature, namely exosomes and ectosomes [2]. They are classified according to their mechanism of biogenesis: the former are of endosomal origin and are released after the fusion of multivesicular bodies (MVBs) with the plasma membrane, while the latter are of plasma membrane origin and either bud from it or are shed directly into the extracellular environment (reviewed in Refs [3–5]). Ectosomes (previously known as microvesicles) can appear as small entities similar to exosomes (around 50–150 nm in diameter), but also as large particles (up to 10 μm), which have been categorized, for instance, as apoptotic bodies, large oncosomes, exophers, free-migrasomes (i.e. those detached from retraction fibers) and, more recently, as extracellular lipidosomes, depending on their origin, mode of formation, state of donor cells and/or their specific composition [2, 6–11]. Unless the precise mechanism of formation is documented, we will refer to them as EVs.

EVs act as mediators of intercellular communication by transferring nucleic acids (e.g., DNA, messenger RNAs, microRNAs), proteins and lipids between neighboring cells or over long distances. They can also “reprogram” the fate of target/recipient cells, for example by promoting their proliferation, and perhaps their differentiation or dedifferentiation, or stimulating their migration, among other cellular processes [10, 12–17]. EVs also play an important role in the immune system as well as during angiogenesis, which are well documented [18–20]. They can also contribute to disease progression, as their aberrant composition and excessive release from unhealthy cells can impact surrounding tissues (reviewed in Ref [21]). This is particularly true for neurodegenerative diseases as well as cancer, where horizontal transfer of nucleic acids or oncoproteins leads to profound phenotypic changes in the tumor microenvironment and promotes formation of the pre-metastatic niche and metastasis [22–27]. Cancer cell-derived EVs may stimulate neo-angiogenesis at the primary cancer site [28–30] and modulate immune responses [31–33]. It has also been hypothesized that they may themselves promote malignant phenotype in secondary sites when the local cells that take them in are predisposed to oncogenic transformation (reviewed in Ref [34]). Furthermore, EVs could promote the invasive activity of cancer (or healthy) cells [35, 36] or the transfer of drug resistance [37, 38] (reviewed in Ref [39]).

Several mechanisms have been described to explain the effect of EVs on target cells or their internalization. These mechanisms are not mutually exclusive, but potentially cell type-dependent. For instance, the attachment of EVs to the cell surface may trigger a cellular response similar to a soluble ligand-receptor interaction, or result in EV-plasma membrane fusion [40–42], or internalization of EVs, which may occur via several processes such as endocytosis, micropinocytosis and phagocytosis [43]. These EV uptake mechanisms have been extensively described and illustrated in numerous review articles [44–50].

Given the wide heterogeneity of EVs [51–53], we will focus in this review on particular EV subpopulations that bear prominin-1 which were originally coined as prominosomes [54]. Over the past two decades, the characterization of the pentaspan membrane glycoprotein prominin-1 (aka cluster of differentiation (CD) 133), and the identification of several interacting lipids and proteins have improved the understanding of the plasma membrane, in particular the molecular organization and dynamics of membrane protrusions [55, 56]. Several studies on prominin-1 have revealed that distinct types of prominent membrane protrusions can serve as source of prominosomes, which are released into various body fluids [11, 54, 57]. Given the diversity of cells expressing prominin-1 (stem cells, terminally differentiated cells and cancer cells) and the variety of physiological fluids containing prominosomes, various functions can be attributed to them. Here, we will describe the different mechanisms of their biogenesis, which may be influenced by prominin-1 itself. We will also discuss the potential role(s) of small and large prominosomes, as they might contribute to the removal of obsolete cellular components and thereby regulate stem cell fate. As carriers of biological messengers, they might also be involved in intercellular communication in healthy and pathological conditions, including cancer.

## Prominin-1

Originally described as a novel cell surface biomarker for neural and hematopoietic stem and progenitor cells (HSPCs) [58, 59], prominin-1 has attracted attention in oncology as it highlights cancer cells with stem cell properties [60–63]. To date (November, 2024), PubMed (National Library of Medicine) lists over 8,000 hits for searches using Prominin or CD133 as keywords. In most of these publications prominin-1 is used as an antigenic marker for the identification, isolation and characterization of cells with stem cell characteristics from various tissues and organs (e.g., brain, kidney, prostate, intestine) of healthy individuals [58, 59, 64–71], or as a potential prognostic biomarker for cancer patients as well as a molecular target for the eradication of cancer stem cells (reviewed in Refs [72–74]).

In addition to its value in biomedicine, prominin-1 aroused particular interest due to its remarkable subcellular localization. It is concentrated in highly curved membranes such as those associated with plasma membrane protrusions, irrespective of the cell type, i.e. stem versus differentiated cells and epithelial versus non-epithelial cells [58, 75]. This specific characteristic led Huttner and his colleagues to name this molecule “prominin”, from the Latin word “*prominere*”, to stand out, to be prominent [58]. Prominin-1 is associated with distinct types of protrusions built on cytoskeleton made up of: (1) actin filaments such as microvilli, stereocilia, microspikes, filopodia and lamellipodia, or (2) a microtubule-based axoneme as found in primary cilia, motile cilia including flagellum of sperm cells or other specialized sensory cilia like the photoreceptor outer segments [58, 75–82]. In these membrane protrusions, prominin-1 is often concentrated at the tip or extremities like the leading edge of lamellipodia [58, 79, 80]. The association of prominin-1 with different cytoskeleton-based protrusions reflects its general preference for cholesterol-rich membrane microdomains in which it binds to membrane cholesterol and possibly organizes them [83].

Prominin-1 is rarely detected in the planar, non-protruding subdomain of the plasma membrane, as shown in vivo in neuroepithelial (NE) cells (i.e. neural progenitor cells) [84], or in vitro when overexpressed in heterologous systems [58, 75, 76, 78]. The expression of prominin-1 in uterine epithelial cells during blastocyst attachment and implantation [85], when these cells lose their microvilli and glycocalyx, increase their cholesterol content and restructure their actin cytoskeleton [86, 87], constitutes a potential exception to its otherwise membrane protrusion-specific localization.

Physiologically, the incorporation of prominin-1 into specific cholesterol-rich membrane microdomains could also explain its association or involvement in various signal transduction pathways [88], which regulate embryonic development, epithelial-mesenchymal transition (EMT), tumor growth and metastasis [89–93]. The molecular and signaling pathways in which prominin-1 has been implicated such as phosphoinositide 3-kinase (PI3K)/Rac-alpha serine/threonine-protein kinase (Akt), Wnt/β-catenin, transforming growth factor-β/Smad, mitogen-activated protein kinase/extracellular signal-regulated kinase (ERK), and Src-focal adhesion kinase have been nicely summarized in recent reviews [94–96].

Structurally, prominin-1 has an unusual membrane topology comprising a N-terminal *e*xtra*c*ellular domain (abbr. EC1), five transmembrane segments separating two small *i*ntra*c*ellular domains (IC1 and IC2) and two large glycosylated extracellular loops (EC2 and EC3) containing around 250 amino acid residues each, and a cytoplasmic intracellular domain (IC3) [58, 97]. Several splicing variants (denoted by the suffix s) have been reported, with an impact mainly on the N- and C-termini (reviewed in Refs [56, 98]). Prominin-1 is also subject to various post-translational modifications such as phosphorylation, ubiquitination, and glycosylation trimming and processing, which can regulate its intracellular trafficking, stability and interactions with other membrane or cytoplasmic proteins (see Ref [96] and references therein). Tyrosine residue 819/828 (numbered according to splice variants s1 and s2, respectively) in the IC3 was found to be phosphorylated by Src and Fyn tyrosine kinases [99]. The latter modification leads to the interaction with the 85-kDa regulatory subunit of PI3K [93], among other proteins – a topic further described below. It should be noted that no specific sequence motif has been identified in prominin-1 that could evidence a potential enzymatic or catalytic activity, and thus highlight its physiological function. Nevertheless, the presence of lipid-binding motifs (e.g., for cholesterol and gangliosides) could explain its involvement in cellular functions dependent on cholesterol-rich membrane microdomains and its incorporation in EVs [96, 100, 101], the latter point being further discussed in the next sections.

## Functional impact of prominin-1 on membrane protrusion-dependent cellular activities

### A look at photoreceptor cells across species

To grasp the involvement of prominosomes in various cellular events, it is essential to have knowledge of the biological characteristics of EV donor cells in a given tissue as well as the potential membrane sources, and the studies of prominin-1 were instructive in this respect. The functional relevance of the affinity of prominin-1 for membrane protrusions has been first demonstrated in the visual system, where prominin-1 is enriched at the base of the rod photoreceptor outer segment – a modified sensory cilium [77]. There, prominin-1 contributes to the biogenesis of the highly curved, cholesterol-enriched membrane outgrowths as precursors of photoreceptor discs [102, 103]. By binding to protocadherin-21 (see below), prominin-1 organizes the nascent precursors of photoreceptive membranes allowing their proper integration into the outer segment cytoplasm [104]. Dominant or recessive mutations in the *PROM1* gene impacting its expression or structure have been linked to different forms of retinitis pigmentosa, macular dystrophy, and cone-rod dystrophy eventually leading to blindness [77, 104–108]. These phenotypes are mimicked in genetically engineered murine models such as those expressing human prominin-1 dominant mutant R373C (e.g., mutation of arginine 373 to cysteine residue in its first extracellular loop) and in various *Prom1* knockouts [68, 104, 109–111], or the spontaneous knockout mouse line *Prom1*^*rd19*^ with a single nonsense mutation (introducing an early premature STOP codon) [112, 113]. In these models, membrane overgrowth and misalignment of new discs were observed in the outer segments of rods and cones, resulting in the loss of photoreceptor cells over time. Interestingly, the phenotypes seen in transgenic mice expressing human prominin-1 R373C mutant are somehow reminiscent of those observed upon depolymerization of actin filaments mediated by cytochalasin D or the knockout of protocadherin-21, which also results in oversized outer segment discs [104, 114–117]. The R373C mutation in prominin-1 also impedes the proteolytic shedding of the extracellular domain of protocadherin-21 [104], an essential step in the outer segment assembly [118]. Protocadherin-21 is a photoreceptor-specific cadherin located at the leading edges of newly formed discs and connecting them with the adjacent membrane of the inner segment, thereby regulating the elongation of precursor discs [119]. These data, together with its preferential association with highly curved membranes, indicate that prominin-1 is involved in the formation of new lamellar discs and the maintenance of the proper structure of the photoreceptor outer segment through its interaction with other proteins and possibly lipids (e.g., cholesterol) (reviewed in Refs [120, 121]). Its expression in the open discs of cone cells [122, 123], nevertheless rules out a direct role in the sealing of new photosensitive disc in the rod cytoplasmic compartment [124].

The expression and influence of prominin-1 on retinal function and photoreceptor morphogenesis have also been demonstrated in non-mammalian species such as *Drosophila melanogaster*, among others [121, 124–129]. The compound eyes of the fruit fly have an open rhabdom system, where the rhabdomere of each photoreceptor cell is separated from the others and acts as an independent light guide for each ocular unit called the ommatidium [130]. Interestingly, the *Drosophila* homologue of prominin-1 was found to be concentrated at the tip of the microvilli-based rhabdomeres that contain the photo-sensitive molecules [131]. By interacting with the secreted protein Spacemaker/Eyes Shut [132], it prevents unwarranted contacts between microvilli of adjacent rhabdomeres, and confers structural integrity to each of the separate rhabdomeres [131] (reviewed in Refs [123, 133, 134]). Remarkably, human prominin-1, when ectopically expressed in the *Drosophila* rhabdomeric *prominin* null mutant is properly sorted in developing rhabdomeres and is able to prevent the inter-rhabdomere adhesion, whereas the expression of its mutated form (R373C) somehow phenocopies the morphogenic alteration observed in ciliary photoreceptors from transgenic mice [104, 135]. Thus, prominin-1 appears to be a key player in the maintenance of the morphological integrity of photoreceptors from insects to mammals in spite of the significantly distinct structural principles of their visual organs [135, 136]. This aspect is particularly astonishing, as the amino acid identity between *Drosophila melanogaster* and human prominin proteins is less than 20% [135, 137]. This suggests that its activity is partially based on its secondary and/or tertiary structure as well as on its specific interacting partner(s), and its operability in actin- or microtubule-based membrane protrusions such as microvilli and cilia. Further details on the role of prominin-1 in photoreceptors and EVs derived therefrom, deduced from data obtained in studies of primary cilia, are discussed below.

### Ciliary assembly, beating and membrane stickiness

Recent studies have highlighted the role of prominin-1 in various physiological processes through its organization of membrane protrusions in cell types other than photoreceptors. For instance, prominin-1 was found to regulate the assembly and disassembly of primary cilia with an impact on stem cell activation as exemplified in the dental epithelium of mouse incisor tooth [138, 139]. There, prominin-1 controls the transition axis that coordinates the transformation of stem cells to transit-amplifying cells by orchestrating the dynamics of primary cilia [140]. In *Prom1*^–/–^ mice this mechanism is disrupted and affects the transcriptional activity (e.g., signal transducer and activator of transcription 3 (Stat3)) in response to Sonic Hedgehog signaling, possibly by altering the initial location of prominin-1 interactor, the transcriptional factor Krüpper-like zinc finger protein GLI-similar 2 (Glis2), in the primary cilium, and thus their co-translocation to the nuclear compartment [140] (reviewed in Ref [141]).

In addition to mammalian prominin-1, silencing zebrafish prominin-3 has been shown to reduce the number and length of motile monocilia in Kupffer’s vesicles [142]. The lack of prominin in this transient organ that is present during teleost embryogenesis results in molecular and anatomical defects such as left-right asymmetry. The latter is caused, at least in part, by a nodal flow mechanism based on coordinated ciliary beating [143]. Alterations in the resulting fluid movement due to ciliary defects might impact an intrinsic mechanosensory stress mechanism and/or the transport of soluble signaling molecules and morphogens embedded into potential carrier vesicles described as nodal vesicular parcels [144–147]. Coincidentally, a similar phenotype (i.e. left-right asymmetry) was observed upon inactivation of the transcriptional factor *nephrocystin-7* (*NPHP7.2*) gene, the zebrafish homologue of Glis2, showing impaired ciliary motility [148]. Thus, prominin-1 seems to be involved in the regulation of the dynamics of primary cilia and, depending on the context, could influence cellular activities, including stem cell proliferation and differentiation. The association of prominin-1, or other members of the prominin family [125, 137, 149], with cilia-mediated cellular processes may have implications for embryonic development, health conditions [150, 151], and a variety of disorders known as ciliopathies [152, 153].

A detailed examination at the ultrastructural level of tissues and organs that express prominin-1 and/or functional assays may reveal a subtle effect of prominin-1 on the structure of protruding membranes. For instance, constitutive knockdown of murine prominin-1 has been shown to have a mild effect on ciliary organization in multiciliated ependymal cells, as dynamic cilia tend to clump together either at their tips or along their length [150]. Not all cilia are impacted. This phenotype is to some extent amplified in the mouse model of drug-induced type 2 diabetes mellitus, where a delocalization of prominin-1 from ependymal ciliary structures results in ciliary tangling and sticking together, particularly at their tips [150]. Entanglement and collapse of ciliary tufts within a given cell or between adjacent cells leads to the formation of ciliary structures oriented in opposite directions [150]. This can result in impaired coordinated movement of these motile cilia hindering correct cerebrospinal fluid (CSF) flow and thereby compromising the transport of signaling molecules and EVs [154]. Increased adhesiveness among prominin-1-deficient ependymal cilia resembles the “stickiness” of rhabdomeric microvilli observed in *Drosophila* eyes lacking prominin homologue [131]. These findings support, albeit indirectly, a potential involvement of prominin-1 in the mechanism underlying ciliary impairment in type 2 diabetes mellitus. It remains to be determined whether another molecule acting as *Drosophila* Spacemaker could interact with prominin-1 in ependymal cilia and provide anti-adhesive characteristics (see Discussion in Ref [150]). Another example of the effect of ablation of the *Prom1* gene on motile cilia was provided in the multiciliated cells present in the respiratory system [151]. Using an air-liquid interface culture of prominin-1-deficient airway cells, it was elegantly demonstrated that the absence of prominin-1 leads to longer cilia that beat at a lower frequency, which negatively impairs the unidirectional mucociliary transport [151]. Such phenotypes could influence the mucociliary clearance and, consequently, the protection level of the lung against inhaled pollutants and pathogens.

Despite the wide range of prominin-1 expression, the phenotypic consequences of prominin-1 deficiency appear to be limited to certain organs, particularly to those lacking its paralogue prominin-2, like the retina [137, 155]. This might also explain why prominin-1-deficient mice are viable [68, 69, 109–111, 156]. Like prominin-1, prominin-2 shows a preferential association with membrane protrusions including those associated with basolateral membranes of polarized epithelial cells, where prominin-1 is absent [80, 137, 157–159]. Although the amino acid sequence identity between both mammalian prominins is low (< 30%), prominin-2 may nonetheless functionally compensate for the absence of prominin-1 as illustrated by its upregulation in adult murine hippocampus in *Prom1*^–/–^ mice [70].

Overall, the information gathered over the years indicates that beyond its value as a marker of stem (and cancer stem) cells, prominin-1 has an impact on various cellular processes involving protruding membrane structures, and perhaps EVs derived thereof (reviewed in Refs [47, 160, 161]. Yet, this contribution remains underestimated.

Hence, the organization and/or functionality of certain types of prominin-1^+^ membrane protrusions and their dynamics, exemplified by the release of EVs, merits further interest as they could affect the fate of the donor cell and/or its communication with surrounding cells within a given tissue or organ. In this respect, pioneering studies on mammalian prominin-1 have highlighted (i) new donor membranes as sources of EVs, like microvilli and primary cilia as well as retracting cell extremities at the entry of the mitotic rounding process, or the midbody at the end of cytokinesis, and (ii) the action of prominosomes on recipient cells, notably those in the cancer microenvironment.

## Discovery of prominosomes – lessons from the cerebrospinal fluid

The CSF is produced by choroid plexus cells in the brain ventricles and circulates into the intracranial and spinal compartments [162]. Initially, prominin-1 was found to be released in association with small and large EVs into the CSF of the murine neural tube lumen during cortical development [54] (reviewed in Ref [163]). A detailed characterization of these EVs using differential centrifugation and immunoelectron microscopy defined different types of prominin-1^+^ EVs. The first class was recovered after ultracentrifugation at 200,000*g* and consisted of small (50–80 nm) electron-translucent vesicles reminiscent of exosomes and small ectosomes [2]. The exclusive localization of prominin-1 at the apical membrane of NE cells [58] and the absence of CD63, a tetraspanin protein highly enriched in exosomes [164, 165], in prominin-1^+^ EVs suggest their ectosomal origin [54]. The second class covered relatively large (0.5–1 μm) electron-dense particles in which prominin-1 immunostaining often appeared as a ring structure [54]. They were recovered after 10,000*g* centrifugation and their origin was later identified as the midbody [57], the narrow microtubule-based structure connecting the two daughter cells at the end of cytokinesis [166]. The identification of the midbody as a source of large EVs defined a new class that is now referred to as secreted midbody remnants [167, 168]. Various post-mitotic functions have been described for them (reviewed in Ref [169]). In sum, prominin-1 not only marks membrane protrusions, but also small and large EVs collectively referred to as prominosomes [54].

## Occurrence of prominosomes in various body fluids

Given the widespread expression of prominin-1 in embryonic and adult tissues, its detection in murine CSF led us to examine other physiological fluids in humans and mice. Marzesco and colleagues were the first to report the presence of prominin-1 in various adult human biological fluids including urine, saliva, seminal fluid and lacrimal fluid [54]. In all of them, prominin-1 was associated with small EVs that were recovered upon ultracentrifugation. In lacrimal fluid, a significant amount of prominin-1 was also detected after low-speed centrifugation (10,000*g*) indicating the recovery of either large prominosomes or aggregates of smaller ones [54]. In saliva, prominosomes differ from those found in the CSF by the presence of CD63 [170]. Human CSF also contained small prominosomes [171]. Interestingly, their concentration is much higher (almost 10-fold) in infants than in adults, with a steep decline after birth up to around 10 years of age. In adulthood, their concentration remains low and varies little from one individual to another. This is in line with the composition of the CSF being constant in adults [171].

As in humans, prominosomes were found in mouse urine showing a good correlation between species with respect to their release [80]. Interestingly, prominin-2 was also detected in human saliva and urine, and mouse urine, in association with small EVs [80, 155, 158]. The ectopic co-expression of prominin-1 and prominin-2 in polarized epithelial cells showed that both proteins were partly associated with the same small EVs released from the apical domain, suggesting a common sorting mechanism [80]. Thus, prominosomes can be assigned to EVs that contain either prominin-1 or prominin-2 (or both). Whether a particular prominosome harbors both molecules has yet to be proven in vivo. Prominosomes released from the basolateral domain solely contained prominin-2 consistent with its localization to both the apical and basolateral domain [80, 158].

Importantly, the tissues associated with body fluids containing prominosomes (prominin-1^+^ or prominin-2^+^) showed corresponding protein expression, e.g., the membrane protrusion-rich cells of various ductal systems [58, 78, 80, 81, 155, 157, 158, 170, 172–177]. The diversity of origins of EVs in body fluids complicates their characterization, particularly when they share a small size (< 150 nm in diameter) and surface proteins such as prominin-1. After the identification of the precise cellular source(s) of a given type of EVs and its defined set of cargoes (proteins, lipids, and nucleic acids), multi-omics data collection could be highly instructive in terms of the physiology of the tissue/organ generating them. For example, the large amount of data provided by liquid biopsies could be helpful for the diagnosis and disease management of cancers at all stages – a topic further developed below [178, 179]. Further research on prominosomes could be of clinical interest, especially because of the relevance of prominin-1 as a stem and cancer stem cell marker.

## Origins and mechanisms of the formation of prominosomes

The selective subcellular localization of prominin-1 in membrane protrusions of polarized epithelial cells was the first indication that these structures can serve as donor membranes for prominosomes, in this case ectosomes [54]. It has been shown that the direct interaction of prominin-1 with membrane cholesterol and its incorporation into a particular cholesterol-rich membrane microdomain lead to its specific retention in these protrusions [76, 83], and are important for their proper organization as well as the release of small prominin-1^+^ ectosomes [180]. However, the intracellular localization of prominin-1 in endosomal compartments of some cells, notably HSPCs and cancer cells [75, 181], suggests an additional source for such small EVs [182, 183]. Hence, prominosomes can be either of ectosomal or exosomal origin.

In the following sections, we will highlight data showing (i) the potential mechanism(s) regulating the subcellular localization of prominin-1 in different types of protrusions and their impact on the release of prominin-1^+^ ectosomes; (ii) the inclusion of prominin-1 in the midbody at the end of cytokinesis and its release thereof in small and large EVs; (iii) the surprising role of prominin-1 on the incorporation of lipid droplets into a new type of large EVs, namely extracellular lipidosomes, and finally (iv) the endocytosis of prominin-1, its intracellular transport in MVBs, and its extracellular discharge in association with exosomes. These topics will be discussed in relation to various interactions between proteins and lipids that regulate these processes.

### Prominosomes derived from microvilli of polarized epithelial cells

*Retention of prominin-1 in microvilli* – One of the hallmarks of epithelial cells is their apical-basal polarity, where the apical and basolateral membrane domains differ from each other in terms of their protein and lipid composition (reviewed in Ref [184]). This polarization can be either achieved through direct sorting and targeting of new components to a specific plasma membrane domain from the trans-Golgi network (TGN), or by an indirect sorting mechanism via transcytosis [185–187]. In addition, randomly delivered membrane proteins could be specifically retained in one domain by interacting with underlying cytoskeletal components and/or the extracellular matrix while being endocytosed (or cleaved/degraded) in the other domain [188, 189]. Moreover, tight junctions contribute to the maintenance of the apico-basolateral polarity of the membrane by restricting the lateral movement and diffusion of membrane components, particularly lipids in the exoplasmic leaflet [190] (reviewed in Refs [191, 192]).

The first evidence that microvilli are involved in the polarized distribution of prominin-1 comes from studies using the polarized epithelial Madin-Darby canine kidney (MDCK) cell line for the ectopic expression of murine prominin-1 [58, 76, 83]. They revealed that its sequence contains dual information important for its specific subcellular localization: (i) for the direct apical sorting from the TGN, and (ii) for the specific retention in apical microvilli [76]. The microvillar confinement of prominin-1 is maintained even when tight junctions are impaired or absent as seen in cells cultured in medium with low calcium content [76, 193] and NE progenitor cells after the transition from the neural plate to the neural tube stages [58, 194, 195]. These observations suggest that microvilli might contribute to the asymmetric distribution of apical components during neurogenic cell division [196]. In *Drosophila* photoreceptor cells, the retention of prominin in rhabdomeric microvilli may also not require any physical barrier in agreement with the fact that none has been detected to date [135]. This might also hold true for the segregation of prominin-1 on the surface of HSPCs, both when morphologically rounded or elongated. In the former state, HSPCs form a microvillar pole, while in the latter they harbor a uropod structure at the rear pole that contains numerous microvilli-like structures [79, 197–200]. Hence, the selective retention of prominin-1 in microvilli contributes to its polarized distribution at the cell surface of epithelial and non-epithelial cells. In contrast, prominin-2 is distributed in a non-polarized fashion in epithelial cells as mentioned above, but still highly associates with membrane protrusions irrespective of the membrane domain or cell types [80, 137, 159].

The specific microvillar localization could be facilitated by the anchorage of prominin-1 to the structural core of microvilli, i.e. the dense bundle of parallel cross-linked actin filaments and cytoplasmic adaptor/membrane binding proteins [201–203]. Yet, the deletion of the cytoplasmic C-terminal region of mouse prominin-1, the domain most plausibly engaged in such interactions, failed to impair its microvillar localization [76]. A similar conclusion was drawn for *Drosophila* prominin in photoreceptor cells [135].

*Association of prominin-1 with cholesterol-dependent membrane microdomains –* Membrane microdomains are unique, stable or dynamic platforms that actively incorporate particular lipids and proteins and exclude others [204]. The best studied membrane microdomains are lipid rafts due to their involvement in a multitude of cellular activities such as membrane trafficking, cellular polarity, signal transduction, pathogen-host interaction among others [88, 205–208]. Lipid rafts are small dynamic and fluid-ordered domains where lipids are more densely packed than the surrounding membrane bilayer [209–212]. They are especially enriched in sterols and sphingolipids in the exoplasmic leaflet. The association of a given protein with lipid rafts is most commonly determined by testing its solubility in the non-ionic detergent Triton X-100 (reviewed and discussed in Refs [213, 214]). If a protein is insoluble in Triton X-100, its incorporation (or association) in lipid rafts is assumed, as shown for the glycosylphosphatidylinositol (GPI)-anchored placental alkaline phosphatase (PLAP) [215]. Although prominin-1 is completely soluble in Triton X-100, its retention in microvilli has been shown to be dependent on membrane microdomains, where prominin-1 is engaged with surrounding membrane components through lateral interactions [83]. Its association with these microdomains was demonstrated by its insolubility in another detergent called Lubrol WX and its separation from soluble components using a buoyant density gradient [83]. The incorporation of prominin-1 in these peculiar microdomains that differ from classical lipid rafts was further supported by immunofluorescent staining of the apical membrane of MDCK cells. It revealed the segregation of prominin-1 and PLAP into microvilli and the planar region of the membrane, respectively [83] (reviewed in Ref [180]). Interestingly, a mild extraction of membrane cholesterol performed in the cold using the cholesterol sequestering compound methyl-β-cyclodextrin (mβCD) resulted in the delocalization of prominin-1 from the microvilli and its colocalization with PLAP [83]. The direct interaction of prominin-1 with membrane cholesterol was demonstrated using a photoactivatable cholesterol analogue [83, 216]. Motifs similar to the linear cholesterol recognition/interaction amino acid consensus (CRAC) sequence (L/V-X_1 − 5_-Y-X_1 − 5_-K/R), and its reverse/mirror (CARC) sequence (K/R-X_1 − 5_-Y/F/W-X_1 − 5_-L/V) [217–220], have recently been described in the transmembrane domains of prominin-1, and could be involved in such membrane lipid interactions [96, 101]. Besides membrane cholesterol, monosialotetrahexosylganglioside (GM_1_) was found to colocalize with prominin-1 in microvilli and primary cilia of MDCK cells as well as microvilli-like protrusions and filopodia of non-epithelial cells [221, 222]. In contrast, monosialodihexosylganglioside (GM_3_) segregates from prominin-1 in membrane protrusions of the apical membrane and localizes to the planar areas similar to PLAP [221]. Taïeb and colleagues proposed that prominin-1 contains two distinct ganglioside-binding sites: a GM_1_-binding site in the EC1, and a GD_3_-binding site near its second transmembrane domain at the extracellular site [100]. These data indicate the existence of distinct subtypes of membrane microdomains that harbor different gangliosides [223].

Lipid rafts and other small microdomains could also be part of, or promote the formation of, larger membrane domains [205, 224–226]. Their stabilization might be based on the formation of cholesterol-dependent supramolecular protein-protein/lipid membrane complexes that involve the cytoskeleton and/or extracellular partners associated with the extracellular matrix or glycocalyx [227, 228]. The anchorage and/or engagement of prominin-1 in such large supramolecular complexes could also limit its lateral diffusion, particularly at the base of the microvilli, where strong membrane curvature could act as a physical barrier. The same may apply to apically located sphingomyelin clusters, whose lateral diffusion is also limited, even in the absence of functional tight junctions [229]. Interestingly, these sphingomyelin clusters are essential for the formation of microvilli [230, 231].

Palmitoylation of integral membrane proteins has been shown to be involved in their association with membrane microdomains [232]. It remains to be assessed whether this particular covalent attachment of lipophilic moieties to prominin-1 could promote its association with membrane microdomains. The conserved cluster of cysteine residues at the boundary of the first transmembrane domain and the beginning of the first small intracytoplasmic loop (IC1) of prominin-1 (or other prominins) might be subjected to such lipid modification (reviewed in Ref [180]).

Overall, prominin-1 is associated with a particular membrane microdomain and, together with other specific constituents (membrane proteins and lipids), may participate in the organization of the plasma membrane, notably specific subdomains such as microvilli or other protrusions.

**Fig. 1:**
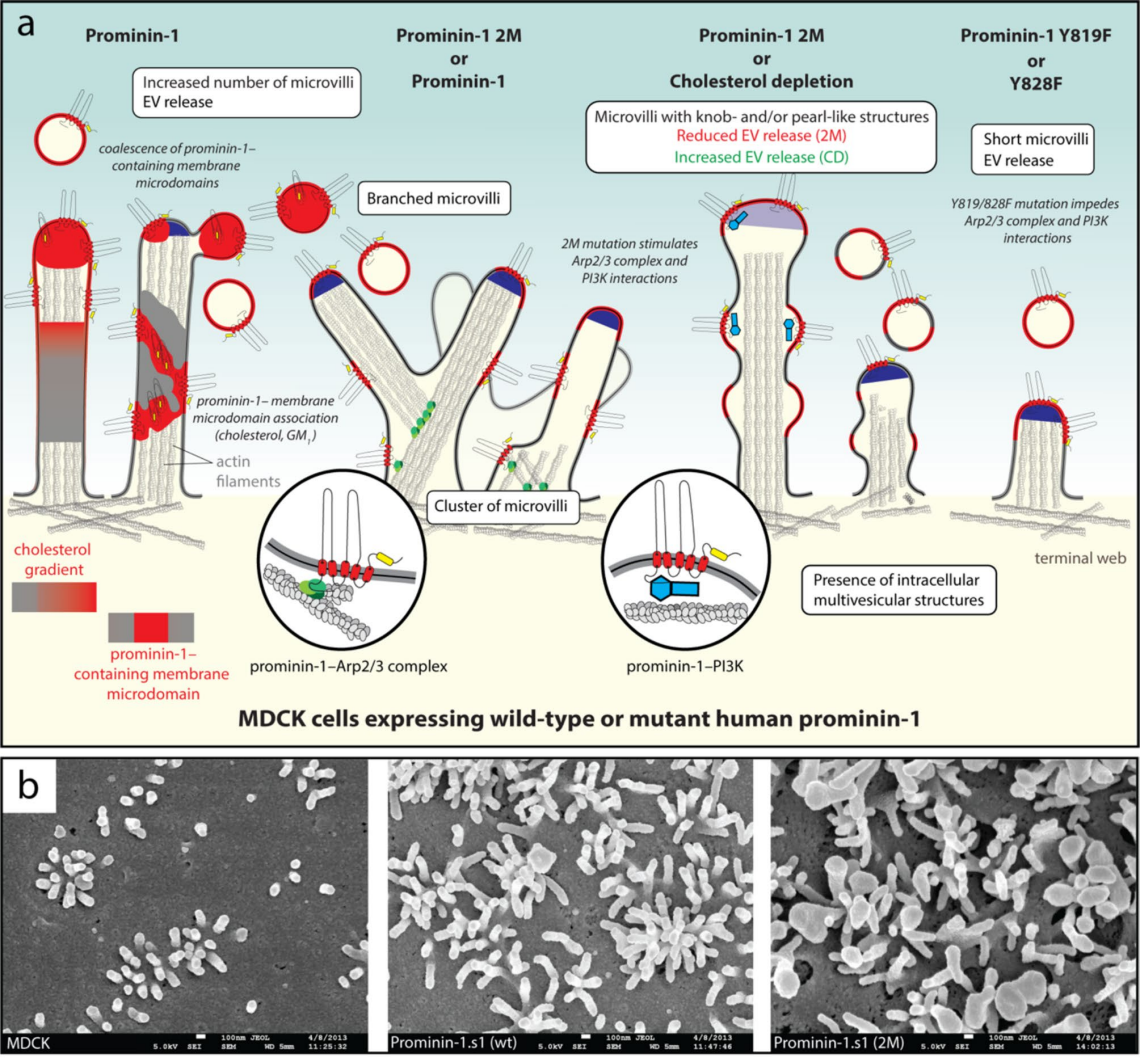
Small prominin-1^+^ ectosomes are released from microvilli on the apical plasma membrane of epithelial cells. **a**,** b** MDCK cells stably transfected with wild-type (wt) or mutants of human prominin-1.s1 splice variant were characterized in terms of morphology and EV release. Their effects on microvilli microvillar architecture and dynamics are illustrated (**a**). Scanning electron microscopy (SEM) micrographs of MDCK cells either non-transfected (MDCK) or expressing prominin-1 wt and the 2M mutant are shown (**b**). The 2M mutation affects the GM_1_-binding site at the extracellular N-terminus of prominin-1 (yellow rounded box), while the Y819F mutation impairs the phosphorylation of tyrosine (Y) 819 (or Y828F in the s2 variant) in the cytoplasmic C-terminal domain. The latter post-translational modification stimulates interactions with the Arp2/3 complex (green) and the PI3K (cyan). Cholesterol-rich membrane microdomains (outlined in red) could be generated either by a gradient of membrane cholesterol towards the tips of microvilli, or by the coalescence of small prominin-1-containing membrane microdomains with both mechanisms leading to the budding of ectosomes (left). Expression of human prominin-1 increases the number of microvilli and leads to branched and/or clustered microvilli, a phenotype stimulated by the 2M mutation, which also promotes the formation of microvilli with numerous membrane constrictions and reduces the release of prominin-1^+^ ectosomes (middle). The “pearling” state is reminiscent of the phenotype observed upon cholesterol depletion (CD) by means of mβCD treatment. The Y819F (or Y828F) mutation results in short microvilli without any increase in prominin-1^+^ ectosome release (right). The main phenotypes (white boxes) and potential causes (in italics) are indicated. Illustration (**a**) and micrographs (**b**) are based on results presented in Refs [54, 83, 200, 221, 234]. For technical details see Ref [200]. Scale bars are indicated

*Release of small prominosomes from microvilli* – Two different scenarios can be envisaged for the release of prominin-1 from microvilli. They involve different membrane components, the reorganization of the underlying cytoskeleton and the concentration of prominin-1 in membrane buds. First, a concentration gradient of membrane cholesterol and prominin-1 could occur towards the tip of a microvillus (Fig. 1a, Prominin-1, left) [58, 75]. There, this may induce a phase separation of the membrane cholesterol/prominin-1-rich microdomain from the surrounding more fluid domains with weakly packed cholesterol. Together with the local curvature, this could result in the fission and release of a microvillar ectosome [180, 233]. Alternatively, the coalescence of small membrane microdomains into larger units along the microvillus may stimulate a fluid phase separation (Fig. 1a, Prominin-1, left). The small Lubrol WX-resistant prominin-1^+^ membrane complexes could be recovered by ultracentrifugation (1 h, 100,000*g*), while the larger domains already sedimented at low speed (10 min, 17,000*g*). In support of the latter scenario, stimulating the clustering of cholesterol or prominin-1 at the cell surface by incubating the MDCK cells with a low concentration of saponin or anti-prominin-1 antibody, respectively, prior to detergent solubilization increased the conversion of small to large Lubrol WX-resistant membrane complexes [83]. Thus, the clustering of prominin-1 molecules and associated membrane microdomains along the microvilli could lead to the formation of a particular region at their sides or ends, where EV budding and release progress (Fig. 1a).

The glycan moieties of prominin-1 could also be engaged in such a process by interacting with other glycoproteins and/or glycolipids in the membrane or extracellular space. Plasma membrane components that carry sialic acid and N-acetylglucosamine residues can be labeled using fluorescently-tagged wheat germ agglutinin (WGA) lectin. Interestingly, such WGA-labeled glycosylated components colocalize with prominin-1 in microvilli and are similarly redistributed to the planar region of the membrane after cholesterol depletion [83]. Glycan-mediated interactions within or between prominin-1^+^ membrane microdomains may also play a role in their packaging into post-TGN carrier vesicles during the intracellular transport [83, 235].

It has been suggested that prominin-1 has the potential to form dimers and/or oligomers [236, 237]. This property of prominin-1 might facilitate the cross-linking of membrane microdomain-associated components, which in turn could induce changes in the tertiary organization of prominin-1 and its phosphorylation status, and consequently influence its interaction with cytoplasmic proteins. The activities of the latter may regulate membrane-cytoskeleton interactions and thereby impact the tension along protruding structures and potentially their hydrostatic pressure. The prominin-1^+^ membrane microdomains on the other hand could influence the intrinsic membrane curvature, thereby weighing on the overall stability of tubular membrane protrusions and their dynamics (see below) [238, 239].

In agreement with the role of cholesterol-enriched membrane microdomains in the formation of microvillar ectosomes, cholesterol reduction by either blocking its synthesis using lovastatin or its sequestration by mβCD enhanced the release of prominosomes in differentiated Caco-2 colorectal adenocarcinoma cells that endogenously express prominin-1 [75, 234]. Furthermore, the cholesterol depletion induced morphological changes of the microvilli [234]. Their tubular appearance was transformed into a pearling-like structure with membrane constrictions spaced along the entire length corresponding to the size of the small EVs (diameter 50–100 nm), suggesting a differential cholesterol extraction effect on non-membrane microdomains where cholesterol is weakly packed (Fig. 1a, cholesterol depletion, middle) [234]. With this remarkable morphology, we might have captured a physiological intermediate state, which might emerge during the release of EVs. Interestingly, when microvilli developed only a single membrane constriction, it was normally associated with the microvillar tip, suggesting that this is the starting point for such a structural transformation. Numerous shorter microvilli were also detected, potentially indicating a reduction in their length resulting from the stimulation of ectosome release (Fig. 1a). These observations are consistent with the release of prominin-1^+^ ectosomes after Caco-2 cell differentiation [54] – a cellular process that is concomitant with the reduction in membrane cholesterol [240] (see next section). In line with the association of prominin-1 with membrane microdomains within small EVs, prominin-1 binds directly to membrane cholesterol embedded therein [234]. The lipidome of small prominosomes derived from metastatic melanoma cells confirmed the enrichment of certain lipids classically associated with membrane microdomains, such as sphingomyelin [241] (reviewed in Ref [242]).

The appearance of EVs in the vicinity of microvilli has been observed in other systems, such as cholesterol-depleted enterocytes derived from porcine small intestinal mucosal explants [243], and duodenal microvilli of chicks fed with a low cholesterol diet [244]. In the latter case, the pearling of microvilli was also noted. Moreover, in artificial lipid bilayer tubes, induction of vesicle fission was observed after cholesterol removal [245], suggesting that budding and fission of small vesicles are controlled, at least in part, by cholesterol levels. In both natural and artificial tubular structures, membrane pearling reflects the balance between tension and curvature [246–249]. In microvilli, lipid components of the inner membrane leaflet such as phosphatidylinositol 4,5-bisphosphate lipids (PtdlnsP_2_ or PIP_2_) and cross-linker proteins like ERM (ezrin, radixin, moesin) and the motor protein myosin-1a can modulate the membrane interaction with the actin filaments and thereby enhance tension, among other physical features. On the contrary, certain processes and/or associated molecular players that negatively affect the membrane–actin filaments interaction could reduce the microvillar tension resulting in a pearl chain-like morphology [250–253]. These interactions and the resulting tension allow a specific membrane curvature, and the transition from a tubular shape to a pearling state reflects the increase in this curvature [254].

Altogether, the release of prominin-1^+^ ectosomes from microvilli appears to be based on a membrane microdomain-driven process in coordination with underlying cytoskeletal components [234, 252]. Similar processes could occur in protrusions at the basolateral membrane where prominin-2 also binds to membrane cholesterol, associates with membrane microdomains and is released into the basolateral milieu in association with small ectosomes [80].

*Effect of prominin-1 on microvilli morphology and the prominosomes derived thereof* – The role of prominin-1 in the microvilli architecture and the release of microvillar ectosomes was further investigated by expressing human prominin-1 or various mutants in MDCK cells [200]. The mutations impacted either its GM_1_-binding site located in the EC1 (2M mutant) or the cytoplasmic tyrosine residue 819/828 (Y819F or Y828F mutant) or both sites (2M Y819/828F). The overexpression of wild-type prominin-1 led to an increase in the number of microvilli and their clustering. A microvilli cluster is composed of independent microvilli that seem to originate from the same root [200]. Branched Y-shaped microvilli were also detected (Fig. 1a and b). These phenotypical changes were observed throughout all microvilli of the apical membrane of a given cell. Mutating the GM_1_-binding site of prominin-1 enhanced these features and further promoted the appearance of knob- and/or pearl chain-like microvilli and numerous intracellular multivesicular structures (Fig. 1a, Prominin-1 2M, middle, b). As mentioned above, pearling of microvilli has been detected upon the disruption of membrane microdomains mediated by cholesterol depletion (Fig. 1a), reinforcing the model that prominin-1-membrane lipid interactions are involved in plasma membrane trafficking and the shaping of membrane protrusions [200, 234]. However, the release of prominin-1^+^ ectosomes differed between both cases, being inhibited by the 2M mutation and stimulated by cholesterol depletion. This suggests that either prominin-1 integrity or its interaction(s) with lipid/protein partners are essential for the proper membrane organization of microvilli, i.e. the ectosome donor membrane (Fig. 1a, Prominin-1 2M/cholesterol depletion, middle). Such assumption is strengthened by the presence of “frozen” buds at the microvillar tips of cells expressing the 2M mutant [200].

Surprisingly, a slight increase in tyrosine phosphorylation of prominin-1 was observed with the 2M mutant, indicating that this post-translational modification is regulated, at least in part, by the binding of prominin-1 to GM_1_. The phosphorylation-dependent interactions of prominin-1 with the actin-related protein 2/3 (Arp2/3) complex, which mediates actin-network branching [255], might explain the appearance of Y-shaped and clustered microvilli [200]. Y-shaped microvilli were observed previously in other systems such as rat epididymis principal cells [256], human conjunctival epithelium in dry eye patients [257, 258], or in other conditions such microscopic enteritis and coeliac disease [259, 260]. They appear, however, to be distinct from those detected in cells lacking cadherin-8, which seem to be formed through a process of microvillus splitting from the tip to the base [261].

The pearl chain-like microvilli phenotype could be due to an increase in PI3K activity as a result of its enhanced interaction with the 2M mutant [200]. As PI3K phosphorylates PIP_2_ to phosphatidylinositol 3,4,5-trisphosphate (PIP_3_) in the inner leaflet, the reduction of PIP_2_ will weaken the interaction of the microvillar membrane with cytoplasmic scaffolding proteins and thus the underlying microfilaments [262]. The formation of branched and knob-like microvilli could also be coupled as the local production of PIP_3_ can indirectly activate Wiskott-Aldrich syndrome protein family verprolin-homologous proteins (WAVEs) [263], which in turn trigger the activation of the Arp2/3 complex [200]. The morphological alterations of microvilli and their clustering were countered by inhibitors of the Arp2/3 complex and PI3K, or by introducing the tyrosine Y819/828F mutation in the 2M mutant that prevents the binding to the aforementioned proteins [200]. These experimental manipulations restored the release of prominin-1^+^ ectosomes. In contrast, cells expressing prominin-1 containing only the tyrosine mutation showed shorter microvilli (Fig. 1a, Prominin-1 Y819F or Y828F, right), which did not result from intensive EV release [200]. These observations are in line with a previous report showing that PI3K inhibition alters the morphological differentiation of Caco-2 cells and reduces the number and length of microvilli [264].

The PI3K activity was also reported to alter microvilli morphology upon insulin stimulation [265], notably the formation of knob-like microvilli [266, 267]. This indicates that a proper balance between PIP_2_ and PIP_3_ is essential for the elongation of microvilli [200]. Therefore, prominin-1 could influence the phospholipid composition of the inner leaflet (PIP_2_/PIP_3_ ratio) via its interaction with gangliosides (e.g., GM_1_) in the outer leaflet, and thereby promote the crosstalk between lipids in the apical membrane. The prominin-1–PI3K axis may be involved in a sequential and/or coordinated process with the CD34-related sialomucin podocalyxin-1–ERM-binding phosphoprotein-50 (EBP50)–phosphoinositide-4-phosphate 5-kinase β axis, which promotes PIP_2_ lipid synthesis, and enhances ERM protein accumulation, resulting in increased microvillus stability [200, 231, 268, 269]. Hence, the spatial and temporal coordination of the phosphorylation of prominin-1 could serve as a biochemical feature that indirectly determines its involvement in tubular membrane organization. By regulating the interplay of PIP_2_- and PIP_3_-lipids with cytoskeletal proteins, prominin-1 might also modulate the budding of EVs thereof [200, 269].

The interaction of prominin-1 with actin remodeling proteins could be instrumental for the formation of membrane ruffles in the early stages of epithelial cell polarization and, subsequently, of clustered microvilli. Interestingly, Y-shaped microvilli could represent intermediate structures for normal, unbranched microvilli, as suggested during the early stages of salamander microvilli regeneration [270]. In this respect, it has been observed by live cell imaging that a subset of nascent microvilli emerges from the base of pre-existing “mother” microvilli before their separation at the end of their growth [271, 272]. The latter are reminiscent of the Y-shaped and/or clustered microvilli observed upon the overexpression of prominin-1. Therefore, prominin-1–PI3K/Arp2/3 complex axes could not only exert an effect on microvilli structure per se, but also contribute to *de novo* microvilli formation, consistent with the increased number of microvilli in prominin-1-overexpressing cells [200].

Interestingly, PI3K and the Arp2/3 complex interact with the multi-interactor scaffolding protein IQ motif containing GTPase-activating protein 1 (IQGAP1), which also binds to WAVE2, insulin receptor substrate p53 (IRSp53) and ezrin, among others. IQGAP1 is known to participate in the formation of branched actin filaments such as those found in membrane ruffles and lamellipodia [273], and thus to organize the remodeling of membrane protrusions [274–280]. Noteworthily, we found that prominin-1 and IQGAP1 were co-immunoisolated from MDCK and Chinese hamster ovary (CHO) cells in a pattern similar to PI3K and Arp2/3 complex (J.K and D.C, unpublished data) [200]. This data indicates a potential association of prominin-1 with a large protein complex that could promotes not only membrane ruffles in migrating cells, but also the formation of the abovementioned clusters of microvilli [200]. Further studies are needed to decipher all prominin-1 interactors in relation to the crosstalk between membrane and cytoskeletal proteins. This information might also shed new light on its role in the biogenesis and maintenance of various types of membrane protrusions, and their role as membrane donors of EVs.

The involvement of prominin-1 is also evidenced in vivo, as in the duodenal brush border membranes of murine cells lacking prominin-1 (Prom1^*rd19*^ model), EV bud-like structures have been observed, albeit rarely, in the basal region of microvilli, as well as fused microvilli [200]. This supports a role for prominin-1 in promoting the release of ectosomes from the tips of microvilli, as well as an anti-adhesive function, as observed in mouse multiciliated ependymal cells and *Drosophila* rhabdomeric microvilli [131, 150].

### Prominosomes derived from other finger-like protrusions and filopodia

*Finger-like protrusions* – The direct incidence of prominin-1 on finger-like protrusions was demonstrated in human HSPCs [200]. Prominin-1 is selectively concentrated in the uropod at the rear pole of migrating cells [197], where numerous finger-like protrusions are present [200]. The uropod harbors various proteins with adhesive properties and may be involved in intercellular adhesion, motility, and intercellular communication upon contact with another cell [198, 281–283]. Interestingly, membrane microdomain-associated GM_1_ is strongly concentrated in the uropod [281, 283], which is in line with its aforementioned co-localization with prominin-1 in various membrane protrusions. Thus, the same microdomain-based retention mechanism might account for the selective concentration of prominin-1 in uropod-associated finger-like protrusions [284]. Remarkably, prominin-1 silencing in human HSPCs abolished these finger-like protrusions, but not the uropod as such, indicating that the general effect of prominin-1 on various actin-based protrusions is conserved [200]. Whether small prominin-1^+^ ectosomes are released from these finger-like protrusions remains to be determined [181].

*Filopodia* – In transfected CHO cells, prominin-1 is strongly enriched in filopodia where it partly co-localizes with the Arp2/3 complex [58, 200]. Its overexpression or certain mutants led to spectacular morphological changes with long (often branched) filopodia induced by a process dependent on phosphorylation of prominin-1 Y819/828 [200]. Similarly, prominin-2 has been shown to induce a network of filopodia-like protrusions containing markers of membrane microdomains, including GM_1_, in CHO cells and human skin fibroblasts [159]. These data reveal similarities in the composition of two different F-actin-dependent structures, i.e. microvilli and filopodia, regarding prominin molecules, membrane microdomains and cytoskeleton-associated proteins. As observed in microvilli, these components could contribute to the biogenesis, maintenance, and dynamics of filopodia. Interestingly, overexpression of prominin-1 in CHO cells also resulted in the appearance of numerous punctate structures in the vicinity of the filopodia, resembling small particles [200]. They could either be EVs derived from an active budding process or membrane fragments left behind during the filopodial retraction. Myosin-1a and vacuolar protein sorting 4B (a component of the endosomal sorting complex required for transport (ESCRT)) are relevant to this process, as they were shown to be actively involved in the scission and shedding of small ectosomes from induced F-actin-enriched filopodial protrusions in response to plasma membrane damage in HeLa cells [285]. The same is true for large filopodia-derived EVs, where a protein, named missing-in-metastasis (MIM), is involved [286].

Intriguingly, Hori and colleagues have reported that prominin-1 can trigger the formation of multiple, long, cholesterol-enriched fibers at the rear of migrating pigmented epithelium cells [287]. These fibers are independent of actin and microtubule polymerization, but required RhoA/Rho-associated coiled-coil containing protein kinase (ROCK) signaling pathway. Five amino acid residues (KLAK) in prominin-1 located at the junction of its last transmembrane segment and the beginning of the IC3 domain were found to be important for the fiber formation [287]. Coincidentally, this sequence is part of a CRAC motif [96], suggesting that binding to membrane cholesterol may stimulate such protrusions. Tyrosine phosphorylation of prominin-1 does not appear to be required, confirming that these unusual protrusions are distinct from those dependent on F-actin. Morphologically, these cholesterol-enriched fibers are reminiscent of the F-actin-containing retraction fibers left behind by migrating cells, and it remains to be determined whether they can generate small EVs, like those known as “retractosomes” [288].

### Prominosomes derived from ciliary structures

*Primary and motile cilia* – Mammalian and non-mammalian prominin-1 and its paralogues are also associated with distinct types of ciliary structures. They have an impact on their morphology and function as illustrated for the retina (modified cilia), the dental epithelium of the mouse incisor tooth (primary cilia) and other systems (motile cilia) [57, 80, 109, 140, 142, 150, 151]. Recent studies shed light on how prominin-1 regulates ciliary architecture and dynamics.

The primary cilium is as an antenna-like sensory organelle that plays an important role in tissue and organ biogenesis during development and in the adult homeostasis by hosting key molecular players of distinct signaling pathways (e.g., Sonic hedgehog, Wnt, Notch) [289–294]. Besides serving as a signaling hub, the primary cilium is an additional source of EVs, as demonstrated by the release of prominin-1^+^ ectosomes from mammalian cells [57]. Interestingly, primary cilia can act as acceptor structures for EVs as well [295–300]. Ciliary ectosomes may contribute to the clearance of obsolete components, participate in the exchange of biological information between cells and can influence the mitotic process.

**Fig. 2:**
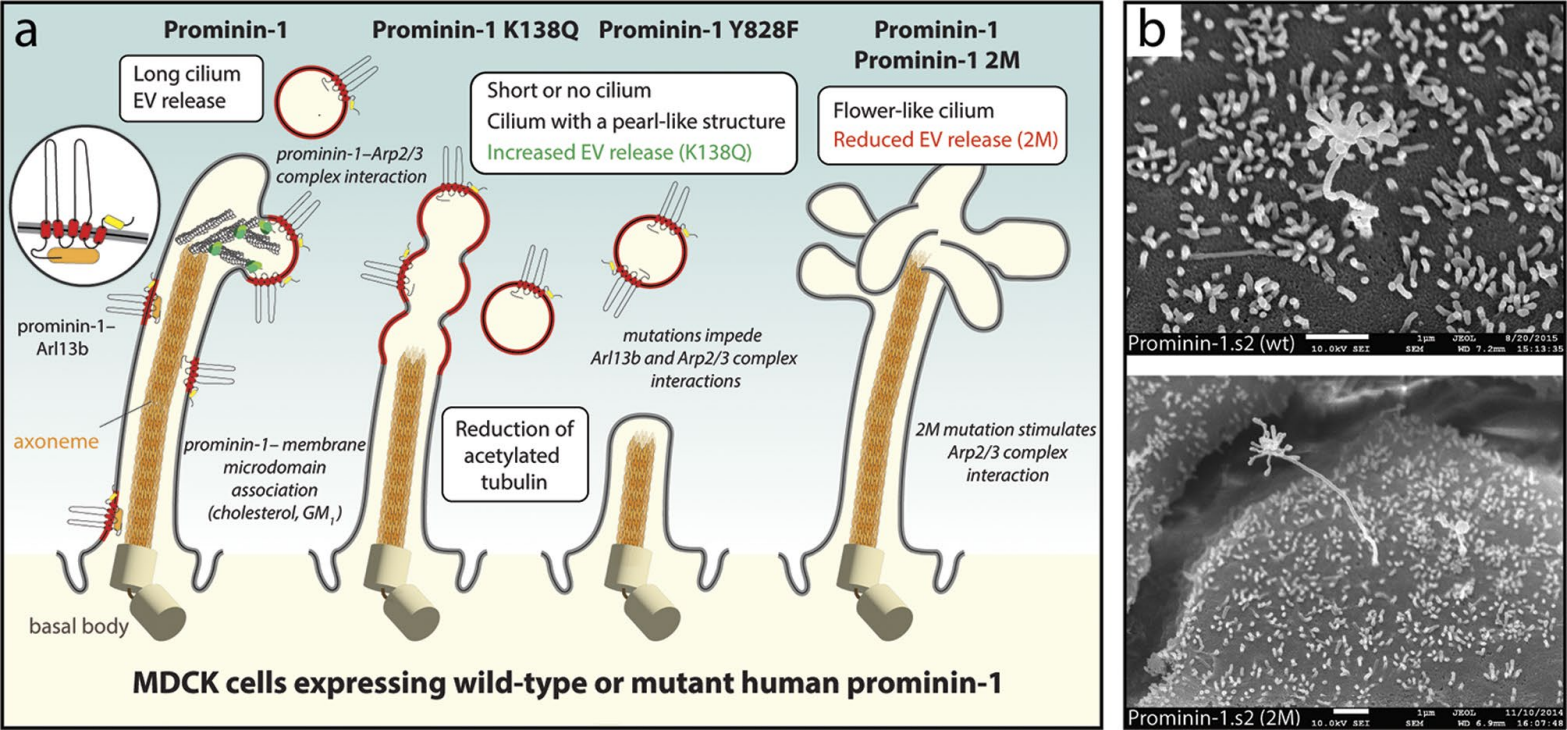
Small prominin-1^+^ ectosomes are released from the primary cilium on the apical plasma membrane of epithelial cells. **a**,** b** MDCK cells stably transfected with wild-type (wt) or mutants of human prominin-1.s2 splice variant were characterized in terms of morphology and EV release. The effects on ciliary architecture and dynamics are illustrated (**a**). SEM micrographs of prominin-1 wt and 2M mutant are shown (**b**). Mutants K138Q and Y828F affect the Arl13b/HDAC6-binding site or the phosphorylation of tyrosine (Y) 828 in the first cytoplasmic loop or C-terminal domain of prominin-1, respectively. The latter post-translational modification favors the interaction with the Arl13b (orange) and Arp2/3 complex (green). Mutant 2M affects the GM_1_-binding site (yellow rounded box) in prominin-1. Cholesterol-rich prominin-1-containing membrane microdomains are highlighted in red, and their coalescence can lead to the budding of prominin-1^+^ ectosomes (left). Expression of human prominin-1 increases primary cilia length, while the K138Q and Y828F mutants reduce cilia length. In the case of the K138Q mutant, some cells show longer cilia with numerous membrane constrictions, and the release of prominin-1^+^ ectosomes is increased (middle). Although rarely, the expression of prominin-1 wt and 2M mutant can create primary cilia with abundant membrane extensions at their tips (right). The main phenotypes (white boxes) and potential causes (in italics) are indicated. Illustration (**a**) and micrographs (**b**) are based on results presented in Refs [57, 140, 142, 221]. For technical details see Refs [140, 200]. Scale bars are indicated

No specific sequence or motif for the association of prominin-1 with the primary cilium or its trafficking into and out of the ciliary compartment, as reported for other ciliary proteins [301–303], has been identified [142]. Its role in the architecture of the primary cilium may rely on its interactions with two key regulators of ciliary morphology, i.e. the ADP-ribosylation factor-like protein 13B (Arl13b) and histone deacetylase 6 (HDAC6) [92, 140, 142]. The small GTPase Arl13b is a member of the Ras superfamily that regulates ciliary length [304–306], while HDAC6 catalyzes the deacetylation of alpha-tubulin and is involved in the disassembly of the primary cilium [307, 308]. Both proteins bind to the lysine (K) residue 138 (numbered in reference to splice variant s2) in the first small intracellular loop (IC1) of prominin-1 [92, 140]. The overexpression of human prominin-1 in MDCK cells resulted in elongated primary cilia compared to wild-type cells (Fig. 2a, left) [140, 142]. In contrast, most of the cells expressing prominin-1 carrying the mutation of lysine to glutamine (Q) (K138Q) displayed short cilia or none at all, accompanied by an increase in the release of prominin-1^+^ ectosome (Fig. 2a, middle). The few remaining longer cilia present in some of these cells showed a pearl chain-like morphology with a shortened axoneme. They might represent an early state in ciliary prominosome release, i.e. before vesicle fission, and the concomitant reduction in cilia length [140]. This prominin-1 mutant as well as the Y828F mutant failed to interact with Arl13b [140, 142]. As observed for microvilli, expression of the Y828F mutant resulted in the reduction of the length of primary cilia without an increase in prominin-1^+^ ectosome release suggesting its phosphorylation is essential for both events (Fig. 2a, middle) [142, 200]. Thus, the interaction of prominin-1 with Arl13b and HDAC6 could affect not only cell fate [140], but also EV-mediated processes (see below). Another candidate for the potential interaction with prominin-1 that might influence primary cilium composition and/or function and, hence, prominin-1^+^ ectosomes derived therefrom, is Glis2/NPHP7 [140]. This protein has been shown to interact with Bardet-Biedl syndrome (BBS) proteins 1, 2 and 11 [148, 309], which are part of a protein complex called the BBSome that is involved in the trafficking of cargo molecules to the cilium [310].

As hypothesized for microvillar structures, the release of ciliary ectosomes may involve the coalescence of specific prominin-1-containing membrane microdomains enriched in membrane cholesterol, and possibly GM_1_ gangliosides [83, 221] (reviewed in Refs [223, 311]). The tyrosine phosphorylation-dependent interactions of prominin-1 with the Arp2/3 complex (and PI3K) might contribute to ciliary EV release (Fig. 2a, left) [200]. Two reports have nicely highlighted the implication of the Arp2/3 complex, or phosphoinositide-dependent F-actin assembly and remodeling, in ectosome release at ciliary tips [297, 298]. The presence of F-actin inside primary cilia was demonstrated [312]. Thus, prominin-1 may mediate the local crosstalk between membrane components and cytoplasmic enzymes depending on its phosphorylation state, and thereby regulating the budding of prominin-1^+^ ectosomes from the primary cilium.

It remains to be determined whether the elongated motile cilia observed in *Prom1*-deficient airway cells are the result of a reduced release of ciliary ectosomes [151]. As the authors mentioned the presence of prominin-1^+^ EVs in the mucus layer, further investigation is needed to understand the correlation of EV release and ciliary length in the respiratory system [151].

*Photoreceptor outer segments* – In photoreceptors, the potential interaction of prominin-1 with the Arp2/3 complex and the resulting remodeling of the branched actin network may be important for the initiation and organization of membrane growth during the generation of photoreceptor discs [313]. The rod-specific knockout of an essential subunit of the Arp2/3 complex was shown to result in a loss of actin filaments, especially at the base of the outer segment, which blocked the formation of new discs. Interestingly, the ciliary supply of membrane constituents was not interrupted and led to the formation of massive membrane outgrowths [314], somewhat similar to the phenotypes observed upon prominin-1 knockout or ectopic expression of the prominin-1 mutant (see above). It remains to be determined whether the potential interactions of prominin-1 with the Arp2/3 complex and protocadherin-21 are sequential or involve large complexes comprising various membrane proteins and lipids.

The association of prominin-1 with F-actin-based processes could reveal a potential correlation between the mechanisms underlying the formation of new discs in the photoreceptor outer segment and ectosome budding from the primary cilium. Although unfrequently detected, the overexpression of prominin-1 (or its 2M mutant showing a significant reduction in the release of prominin-1^+^ ectosomes) [200] occasionally led to the formation of primary cilia with numerous membrane outgrowths at their tips (Fig. 2a, right, b). These observations suggest that prominin-1 can initiate the growth of membrane vesicles and supply them with membrane components, in both types of ciliary structures, which under certain conditions fail to release prominosomes. Interestingly, the inactivation of peripherin (also known as peripherin 2), a photoreceptor-specific tetraspanin membrane glycoprotein, resulted in the secretion of ciliary ectosomes, suggesting that the photoreceptor discs are formed by the peripherin-dependent suppression of ciliary ectosome release [315, 316]. Under native conditions, a small proportion of developing photoreceptor cells were found to release rhodopsin-bearing ectosomes from ciliary tips at the onset of disc morphogenesis, possibly representing a subpopulation of cells with delayed peripherin expression [315]. Selective expression of peripherin at the “close” edge of nascent rod discs could favor the suppression of EV release and allow the initiation of new membrane outgrowth, followed by membrane flattening, alignment and disc elongation, in which prominin-1 and its interacting partners, i.e. membrane cholesterol, Arp2/3 complex and protocadherin 21, may play a prominent role. Clearly, the implication of multifunctional prominin-1 in these intricate cellular processes at the base of the photoreceptor outer segment needs to be further studied [317].

*Sperm tail* – Another ciliary structure where prominin-1 was detected is the tail of mouse spermatozoa, notably those found in the seminiferous tubules of contorted testes, suggesting that it may play a role in spermiogenesis [78, 318, 319]. In the epididymis, its very low expression in sperm cells is associated with the midpiece and the cytoplasmic droplet (also known as Hermes body), when the latter are present [176, 318], implying that this cholesterol-binding protein could be involved in sperm cell remodeling, particularly along the flagellum, where the cytoplasmic droplet and/or small EVs derived from it could be expelled [320–323]. These observations raised the question of whether prominin-1 is essential for fertility. To date, most studies on various models of *Prom1* null mice reported no problem with male fertility [68, 109, 111, 200, 324, 325], with two exceptions. One study noted compromised spermatogenesis in some of the males despite no general interference with development or fertility [156] and the other reported male infertility and structural defects in spermatozoa, with abnormal morphology and reduced motility [326]. Whether these discrepancies reflect the genetic background of the animals, or perhaps other factors, is a matter for further explored [327, 328]. To our knowledge, no patient with retinal dystrophy caused by mutations in the *PROM1* gene has been reported to suffer from infertility [329].

Prominin-1 is highly expressed in the F-actin-based stereocilia of epididymal cells and is associated with small EVs in seminal fluids [54, 78]. It remains to be determined whether prominosomes behave like exosome-like epididymosomes (or other ectosomes) [330] and contribute to the transfer of biomaterials from epididymal cells to spermatozoa, and thus participate in sperm cell maturation [331–334]. The expression of prominin-1 in various other epithelia of the murine male reproductive system raises the issues of its release in association with EVs and of whether these prominosomes play a role in the acquisition of sperm motility, among other things, as has been suggested for prostasomes [335, 336]. The same applies to EVs positive for prominin-2, whose expression has been documented in the male reproductive system [157, 176, 337], as well as in epididymosomes and prostasomes [338, 339].

In sum, the influence of prominin-1 on the architecture and function of structurally different cellular protrusions suggests that the mechanism(s) that regulate their organization and dynamics, including the release of prominosomes coordinated by prominin-1 and its interactors, is (are) conserved.

### Midbody and prominosomes derived therefrom

**Fig. 3:**
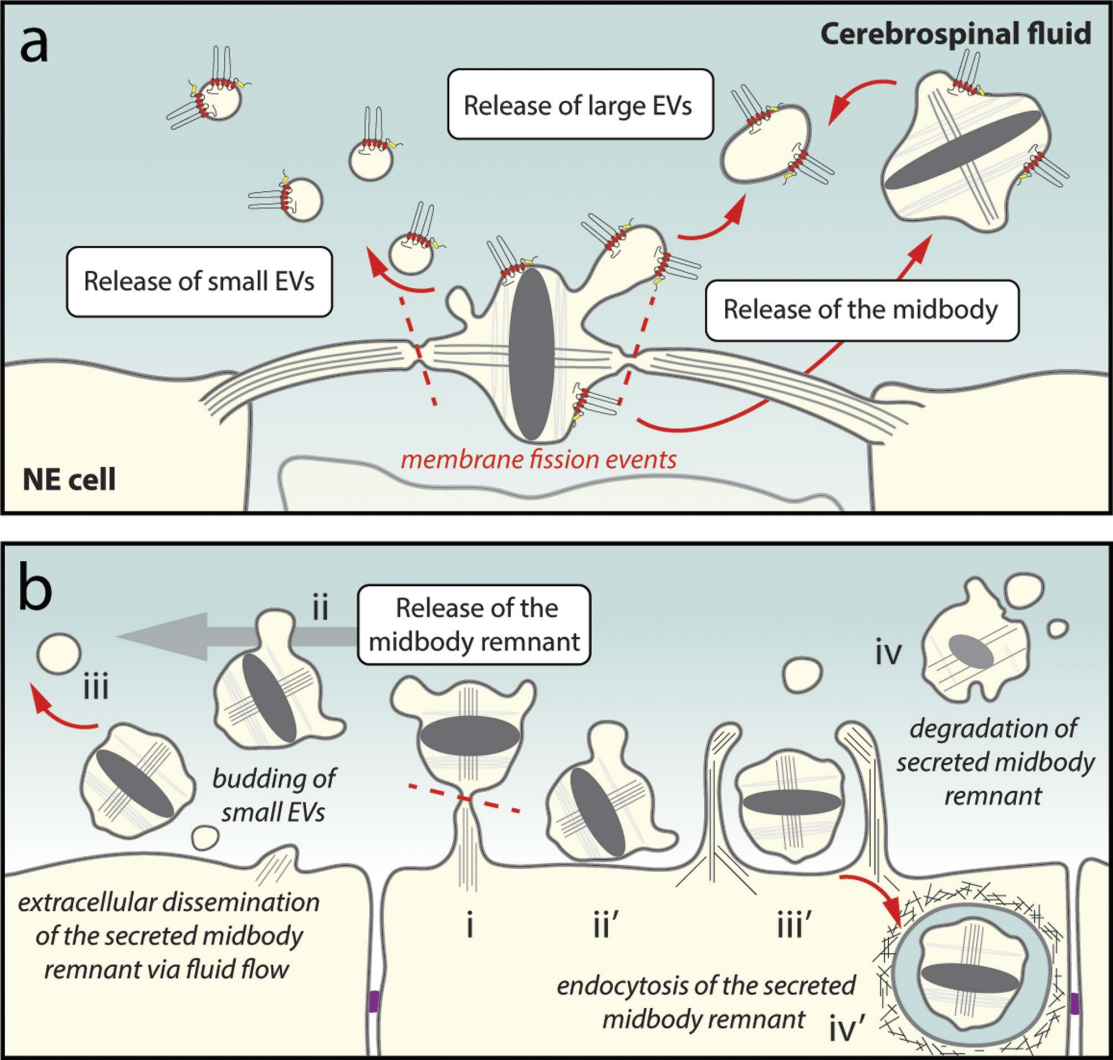
Dividing neural progenitor cells release the prominin-1^+^ midbody and small ectosomes derived therefrom. **a** Various EVs are released into the CSF during brain development. In neuroepithelial (NE) cells, small ectosomes (prominosomes) may originate from the central part of the midbody, where prominin-1 is concentrated in nascent buds. Large prominin-1^+^ectosomes lacking tubulin and anillin, an actin-binding protein associated with the contractile ring of the midbody, are also released. Bilateral membrane fissions on both flanks of the central part of the midbody (red dashed line) lead to its release as secreted midbody remnant and constitutes another source of large prominosomes containing tubulin and anillin. **b** Various fates of the secreted midbody remnant. After a second abscission-like fission (step i, red dashed line), the midbody remnant is released into the brain’s ventricular system and can be transported through the CSF flow to distant cells (ii). Potentially, small and/or large prominosomes continue to bud from the secreted midbody remnant (iii), and/or its extracellular degradation could also occur (iv). Alternatively, the secreted midbody remnant can remain near the cell surface (ii’) or be endocytosed by a phagocytosis-like process (iii’). Actin coats around the endocytosed secreted midbody remnant might slow down its degradation (iv). The midbody could be internalized by distant cells after transport via the CSF. The main events (white boxes) and the potential fate of a given EV (italics) are indicated. Illustrations are based on results presented in Refs [57, 343, 349, 350, 354, 355] and adapted from Ref [55]

By studying the cell biology of NE cells, Dubreuil and colleagues have demonstrated that the microtubule-rich midbody that appears at the end of cytokinesis can be a source of small and large prominin-1^+^ ectosomes (Fig. 3a, see legend for further details). In addition, the central part of the midbody (also called Flemming body) can be fully released into the extracellular space [57]. Although some authors thought that the midbody could be discarded upon cell division [340–343], the common theory about the fate of the midbody involves its inheritance by one of the daughter cells at the end of cytokinesis and its subsequent degradation [344–347]. Both phenomena may co-exist within a given developing tissue and, they can be specific for a particular cell population in culture [57, 348–350]. These conflicting observations might be caused by the difference in cellular state (proliferation versus differentiation) and/or the diverse nature of cells (stem cells versus cancer cells). The association of prominin-1 with the midbody at the end of cytokinesis in NE cells is not necessarily a general phenomenon, as such inclusion is not observed in dividing CD34^+^ HSPCs or in glioma cells [351–353].

The mechanism underlying the inclusion or enrichment of prominin-1 in the midbody of NE cells remains to be identified, but we can speculate that its association with a specific membrane microdomain might also be involved in such process [83, 356]. As the cleavage of the midbody is required at the final mitotic step, the players of the abscission machinery, notably the ESCRT machinery may be implicated [357]. The two interacting partners of prominin-1, tumor susceptibility gene 101 (TSG101) protein and IQGAP1 [200, 358], which are involved in cytokinesis together with centrosomal protein 55, among others, might be engaged [359–362]. Likewise, the interaction of prominin-1 with syntenin-1 [170], a PDZ (PSD95, DIgA, Zo-1)-domain-containing adaptor protein, which in turn binds syndecan and ALG-2-interacting protein X (ALIX) [363], could contribute to the sorting of prominin-1 in the midbody [357]. However, prominin-1 appears to be dispensable for the release of the midbody, as this phenomenon is not impaired in prominin-1-deficient mice [57].

### Extracellular lipidosomes: a new type of large prominosomes

**Fig. 4:**
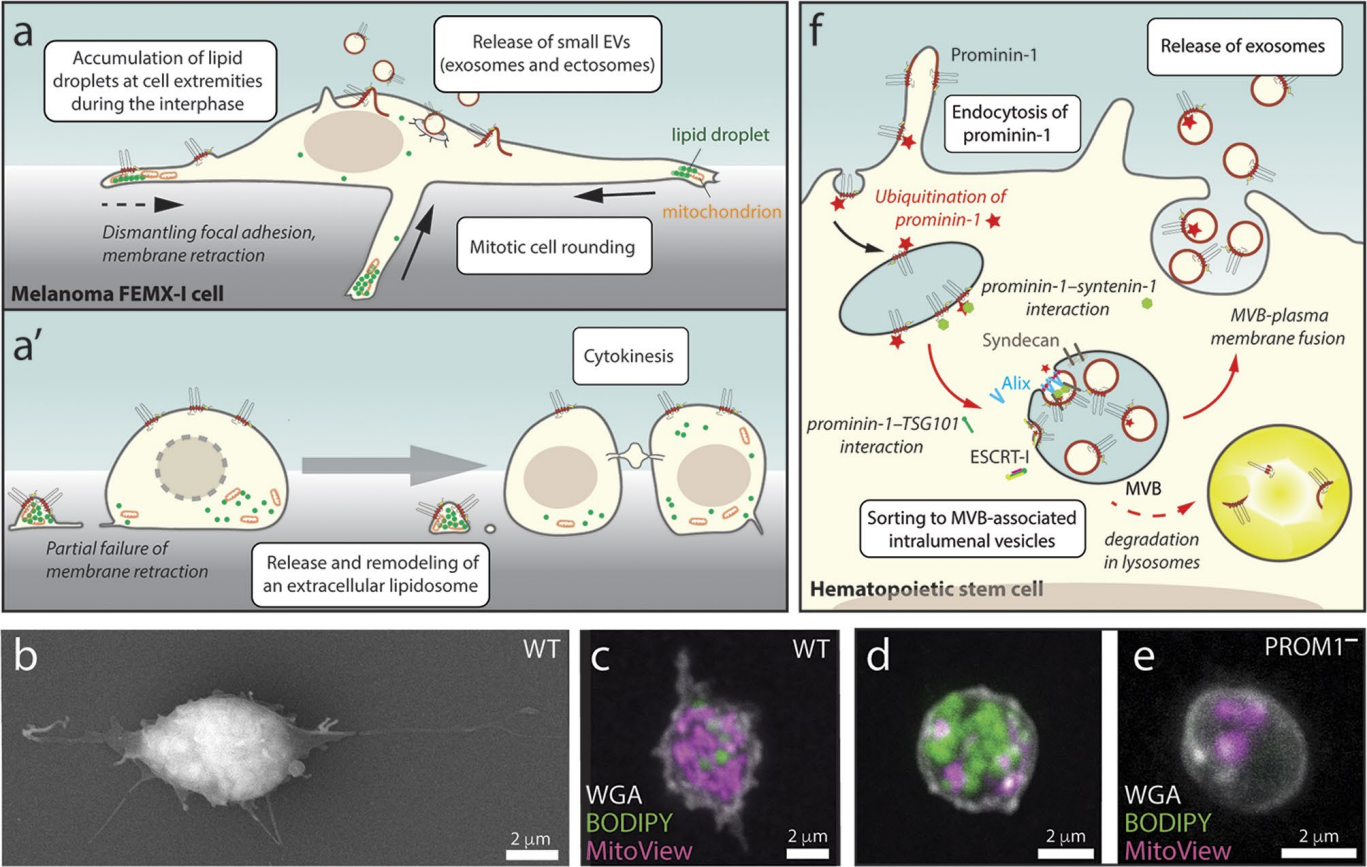
Prominin-1 is released into the extracellular milieu in association with various types of prominosomes. **a**,** a’** In FEMX-I melanoma cells, prominin-1 is distributed over the cell surface during interphase, while the cell extremities contain mitochondria and are enriched in lipid droplets. Small prominosomes are released as exosomes and/or ectosomes and display all characteristics of cholesterol-rich membrane microdomains (red segment). As the cell enters mitosis, the rounding process leads to the retraction of their extremities involving the disassembly of focal adhesion. In some cases, the retraction of a cell extremity is not fully accomplished (dashed arrow) resulting in the release of large prominosomes containing lipid droplets and mitochondria. The so-called extracellular lipidosomes can be taken up by other cells (not illustrated) or may contribute indirectly to the asymmetric distribution of certain organelles between daughter cells. **b-d** Micrographs of extracellular lipidosomes observed either by SEM (**b**) or confocal laser scanning microscopy after triple staining of wild-type (WT) melanoma cells with fluorescently conjugated WGA to highlight glycoconjugates and the fluorescent dyes BODIPY™ 493/503 and MitoView™ Fix 640, which label lipid droplets and mitochondria, respectively (**c**,** d**). **e** Silencing of prominin-1 (PROM1^–^) leads to a redistribution of lipid droplets from the cell extremities to the perinuclear regions (not shown), accompanied by a reduction or complete loss of lipid droplets on the resulting large EVs. The distribution and number of mitochondria are unaffected. **f** In hematopoietic stem and progenitor cells (or cancer cells), prominin-1 is endocytosed en route to late endosomes/MVBs, which will either fuse with the plasma membrane to release their prominin-1^+^ ILVs as exosomes, or fuse with lysosomes and initiate degradation. The ubiquitination of prominin-1 and its interactions with TSG101 or syntenin-1 (and their interacting partners such as syndecan, ALIX or other proteins of the ESCRT-1 or ESCRT-III complex) may contribute to the sorting of prominin-1 into ILVs. The main events (white boxes) and potential causes (in italics) are indicated. Illustrations are based on results presented in Refs [11, 170, 181, 241, 358, 373]. Scale bars are indicated

A new mechanism for the formation of large prominosomes was discovered in a recent study analyzing the cell division of aggressive melanoma cells (FEMX-I) [11], a cell line derived from a lymph node metastasis [364, 365]. These prominin-1^+^ cells display an elongated, bi/tripolar fibroblastic morphology and during the initial phase of cell division, they become rounded [366], a process that involves the disassembly of focal adhesions, retraction of cell margin, and an increase in intracellular hydrostatic pressure due to water influx [367–370] (reviewed in Refs [371, 372]). Interestingly, the incomplete retraction of the cellular extremities, where lipid droplets accumulate [11, 373], triggers the shedding of large prominosomes (2 to 6 µm in diameter) (Fig. 4a, a’), in which lipid droplets are trapped (Fig. 4b-d) [11]. Because of the lipid droplet content, this new type of large EVs has been referred to as extracellular lipidosomes [11].

The inability to fully disassemble certain focal adhesions at the cell extremities could be part, with retraction force, of the mechanism explaining the incomplete cell retraction, and hence the shedding of extracellular lipidosomes. Specific integrins may be responsible for that. Clusters of integrins (referred to as integrin-rich contacts) are known to maintain firm attachment to the extracellular matrix despite the disassembly of peripheral proteins of the focal adhesion at the entry of mitosis [374]. These integrin-dependent persistent mitotic cell adhesions play a role of molecular memory, with new focal adhesions reforming at the same position after cytokinesis. In this respect, it has been highlighted that cell extremities and derived extracellular lipidosomes contain the integrin αVβ3 which appears in small clusters, suggesting that it may contribute to the biogenesis of these large EVs [11]. In lymph nodes, the tight interaction of integrin αVβ3 with vitronectin could favor the release of extracellular lipidosomes from metastatic melanoma cells, and perhaps their cellular uptake [375]. Small prominosomes derived from the same melanoma cells also harbor integrin αVβ3 [241], suggesting its role in the crosstalk between cancer cells and host or targeted tissue. In breast cancer cells, this integrin associated with small EVs binds to the extracellular matrix, facilitating the EV uptake by non-tumorigenic cells [376].

The presence of mitochondria, in addition to lipid droplets, in these large prominosomes is highly interesting as these two organelles interact in synergy, the mitochondria being the energy factory and the lipid droplets the energy reservoir [377–379]. In cancer, the accumulation of lipid droplets has been associated with tumor aggressiveness, and resistance to chemotherapy [377, 380–382]. Suppression of lipid droplet biogenesis has been shown to be sufficient to disrupt cell cycle progression and slow melanoma growth, suggesting that melanoma cells with enhanced lipid droplet capacity are at a metabolic advantage [383]. It remains to be evaluated whether the selective release of extracellular lipidosomes from one extremity of the cell could contribute to an asymmetric distribution of lipid droplets, mitochondria or other organelles between daughter cells, and hence impact their fate (Fig. 4a’) [384–386]. This mechanism would be consistent with the asymmetric distribution of prominin-1 observed in various mitotic stem and cancer cells [196, 351–353]. Furthermore, the release of extracellular lipidosomes may not only modify the metabolism of the donor cancer cells, but also the one of other cancerous or healthy cells after they have taken them up [11]. Thereby, extracellular lipidosomes can influence the cancer cell microenvironment as reported for small EVs [387, 388]. Of note, extracellular lipidosomes do not appear to be involved in the discharge of stressed or damaged mitochondria under stress conditions [11], as has been observed for other large EVs such as exophers and migrasomes [9, 389].

In addition to being a marker of extracellular lipidosomes, prominin-1 has been shown to be indirectly involved in their formation. The silencing of prominin-1 leads to a re-localization of lipid droplets from the cell extremities to the perinuclear region, resulting in a significant reduction in number and eventual total absence of them in these large EVs (Fig. 4e) [11]. It might be of interest to further dissect the effect of prominin-1 on lipid droplets, notably their concentration, as a relation between them and cancer stem cell properties was shown not only in melanoma cells [11, 373, 390, 391], but also in colorectal cancer stem cells [392]. Yet, it remains to be determined whether certain signaling pathways and/or cellular processes are directly involved in this relationship (reviewed in Refs [393, 394]).

Extracellular lipidosome-like EVs are not unique to melanoma cells as similar entities were previously described to be shed directly from the surface of astrocytes [395]. Although prominin-1 expression in astrocytes has been previously documented [82, 324, 396], its association with these astrocyte-derived, large lipid droplet-containing EVs will require further examination. While the release mechanism differs from that of extracellular lipidosomes, their shedding by astrocytes underlines their importance for cells in general. Many questions regarding extracellular lipidosomes remain to be addressed in future studies: (i) their generation and release by other cancer cell types; (ii) whether specific signaling molecules (e.g., chemokines) are involved in attracting recipient cells to the extracellular lipidosomes, as has been demonstrated for free-migrasomes [397, 398]; (iii) the mechanism of their uptake, and (iv) their effect on recipient cell metabolism.

Altogether, in addition to being released in association with the midbody and EVs derived therefrom at the end of cell division by NE cells, prominin-1 can also be released at the onset of cell division in association with extracellular lipidosomes by other cells.

### Prominosomes derived from multivesicular bodies

Beyond its presence on the cell surface, prominin-1 has been detected in intracellular compartments such as endosomes and the Golgi apparatus of various cancer and non-cancer cell lines (e.g., Caco-2, Huh-7, SK-N-DZ, FEMX-I, ARPE-19 cells), and tissue samples [75, 92, 181, 373, 399–401]. These observations suggest that prominin-1 shuttles between the plasma membrane and the endosomal system. Concomitantly, a role for prominin-1 in endocytosis via a cholesterol-dependent clathrin pathway has been proposed [402]. Intracellular trafficking of prominin-1 has been monitored in human HSPCs, where it partially colocalizes with early and/or signaling endosomes [181]. Nevertheless, the bulk of intracellular prominin-1 colocalizes with CD63, suggesting its presence in small intralumenal vesicles (ILVs) – the precursors of exosomes – associated with late endosomes/MVBs [181]. This may account for the release of prominin-1^+^ exosomes through MVB fusion with the plasma membrane, while the fusion of MVBs with lysosomes could lead to degradation of endocytosed prominin-1 (Fig. 4f).

Mechanistically, the (mono)ubiquitination of the lysine residue 848 (K848) located in the cytoplasmic C-terminal domain (IC3) of prominin-1 regulates its incorporation into MVBs by interacting with TSG101 [358]. TSG101 is a well-known component of the ESCRT-I machinery that is involved in the formation of MVBs (Fig. 4f) [403, 404]. The mutation of this lysine to an arginine (K848R) has been shown to impede prominin-1–TSG101 interaction and reduce the release of prominin-1^+^ exosomes, but does not affect its degradation by the lysosomal pathway [358]. The interaction of prominin-1 with syntenin-1 [170], which regulates together with two other proteins (ALIX and syndecan) the sorting of cargo proteins and the intraluminal budding of endosomal membrane [183, 363], may be involved as well (Fig. 4f). It is interesting to highlight that mutations of the prominin-1 GM_1_-binding site described above (mutant 2M) lead to an increased ubiquitination of prominin-1 and stimulate its interaction with syntenin-1, two events that correlate with the presence of numerous intracellular multivesicular structures (see above Fig. 1) [200]. It remains to be determined whether syntenin-1 binds directly to the PDZ-binding motif located at the C-terminal domain (IC3) of certain splice variants of prominin-1 [98] or to ubiquitin molecule(s) linked to prominin-1 as demonstrated for other proteins [405, 406]. The possibility that the interaction between syntenin-1 and CD63 could potentially contribute to the sorting of prominin-1 in MVBs is not excluded [403], as it has been proposed that CD63 interacts with prominin-1 in a membrane yeast two-hybrid screen [92]. At the current stage, we cannot rule out that prominin-1, CD63 and syntenin-1 may be part of a larger protein complex that is sorted into MVBs.

Prominin-1^+^ exosomes are enriched in cholesterol and sphingomyelin [181], suggesting a potential role of specific cholesterol-rich membrane microdomains in the selective protein sorting in ILVs within MVBs. It is perhaps more than a coincidence that CD63 has recently been shown to play a central role in cholesterol sorting in such ILVs which, once released as exosomes, can contribute to the intercellular transfer of membrane cholesterol [407]. Overall, these observations point to the presence of a sorting membrane platform consisting of specific proteins and lipids that interact with each other. Other lipids, like ceramide, have been shown to trigger the budding of vesicles into the lumen of MVBs [408]. The presence of the ganglioside GD_3_ in exosomes and a potential GD_3_-binding site in prominin-1 [100, 409] raises the possibly that this ganglioside contributes incorporation of prominin-1 into MVBs. Hypothetically, prominin-1 could switch between GM_1_- and GD_3_-based membrane microdomain and thereby modulating its affinity for highly curved membranes and/or its function as an organizer of them. Thus, several distinct pathways coexist, and perhaps cooperate, in the intra-endosomal membrane transport of prominin-1 leading to its incorporation into ILVs/MVBs. Whether they are cell type-dependent and/or are influenced by physiological or pathological conditions, and result in the formation of different MVB populations, and hence distinct exosomes, remains to be assessed [410].

Therefore, prominosomes can be released as exosomes after intracellular trafficking in the endosomal compartment, or as ectosomes when they originate from the plasma membrane. As most cells bear microvilli or other finger-like protrusions, both types of prominosomes can be released from a given cell, and hence be present in certain body fluids. Its dual expression in the intracellular compartments and on the surface of HSPCs or differentiated Caco-2 cells represent a good example of cells that can generate both exosomes and ectosomes containing prominin-1 [75, 79, 181, 197, 198, 200]. All these cellular processes are not necessarily exclusive, particularly if the intracellular transport is regulated, e.g., prior to cell division or after cell confluence and/or differentiation, which could explain the presence of prominin-1 in CD63^+^ and CD63^−^ EVs [54, 170, 352, 411]. Since body fluids are in contact with different cell types, they may contain a diverse set of prominin-1^+^ ectosomes and exosomes. For example, a specific fraction of prominosomes derived from saliva, could be of endosomal origin as the associated prominin-1 is ubiquitinated and they are positive for CD63 and syntenin-1 [170].

Further characterization of immunoisolated prominosomes, including identification of other components and subsequent determination of their colocalization with prominin-1 using single-molecule super-resolution microscopy of individual EVs, could help to pinpoint the origin of prominin-1 in a given body fluid [241, 412].

## Role of prominin-1 in prominosome biogenesis

**Fig. 5:**
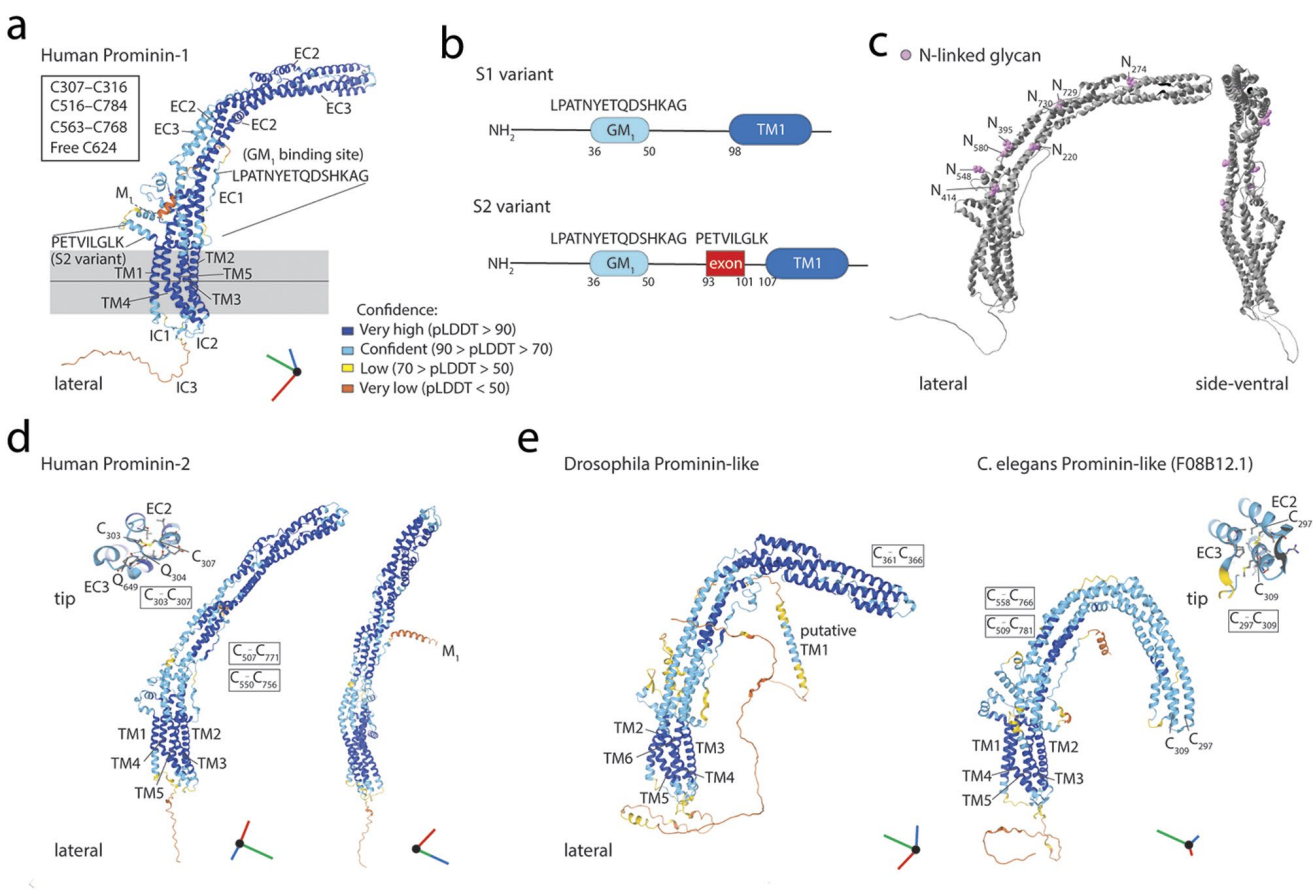
Banana-like shapes of prominin proteins predicted by AlphaFold. **a-e** Models of mammalian prominin-1 (**a-c**) and prominin-2 (**d**), and *Drosophila melanogaster* and *Caenorhabditis elegans* homologues (**e**) are displayed. A lateral view human prominin-1.s1 (UniProt number 043490) is presented with its five transmembrane (TM) domains, three extracellular (EC) and intracellular (IC) domains. The predicted disulfide-bridges formed between thiol groups of cysteine (C) residues are indicated. The sequence and position of the potential GM_1_ ganglioside-binding site (GM_1_, cyan rounded box) and a facultative small exon (exon 4, red box) present in the s2 splice variant, but absent in s1 variant, are shown. This small 9-amino-acid residue encoding exon is located between the potential GM_1_-binding site and the first transmembrane segment (TM1, blue rounded box) of prominin-1 (**b**). The position of all asparagine (N) residues found in the consensus N-glycosylation site (Asn-X-Ser/Thr-Y sequons, where X and Y ≠ proline residue) are indicated (**c**). The predicted tertiary structure of prominin-2 (Q8N271) is very close to that of prominin-1 (**d**). The non-mammalian prominin-like protein found in *Drosophila melanogaster* (P82295) and *Caenorhabditis elegans* (Q19188) also exhibits a banana-like shape, both displaying a stronger curvature than the mammalian ones (**e**). Note that an additional transmembrane segment (TM1) is predicted for the *Drosophila* prominin-like protein in place of a signal peptide, suggesting that this concave side of prominin will face the cellular membrane. Illustrations are based on data presented in the AlphaFold Protein Structure Database [417, 418]

The association of prominin-1 and its paralogues with membrane structures exhibiting high curvature [235] and its activity in modulating their organization irrespective of the presence or type of cytoskeletal structures [58, 140, 200, 287], might be more than coincidental. The intrinsic structural properties of prominin-1 (Fig. 5) in combination with its direct interactions with membrane lipids may have a physical impact on (i) the thickness and fluidity of the membrane bilayer along the curved protrusions, and (ii) the active process of EV budding [239, 413]. The latter may be further stimulated by prominin-1-interacting cytoplasmic proteins that regulate the composition of the inner membrane leaflet and the organization of the cytoskeleton. Prominin-1 and its associated anisotropic membrane microdomains may thus contribute to the overall stability of curved tubular membrane protrusions as structural units, along with the actin- or tubulin-based cytoskeleton [239]. The local membrane deformation that is potentially induced by a fluid phase separation and the reorganization of the underlying cytoskeleton may in fact drive the formation of transient spherical prominences as precursors of ectosomes. These hypotheses are in line with the ability of GM_1_ to self-organize into small nanodomains, and thus initiate positive membrane curvature [414–416]. The dual interactions of prominin-1 with membrane cholesterol and GM_1_ (either simultaneously or singly) may not only affect the cytoskeleton via the activity of Arp2/3 complex and PI3K, but also the membrane organization itself.

At the molecular level, prominin-1–lipid interactions [83, 100, 200], and perhaps prominin-1–prominin-1 interactions [236, 237], might coordinate the architecture of membrane protrusions. According to the banana-shaped model proposed for prominin-1 by the artificial intelligence program AlphaFold [417, 418], its two large extracellular loops (EC2 and EC3) are closely intertwined. The GM_1_-binding site in the EC1 domain is located on the concave side of the protein, i.e. in an exposed position that supports the interaction with gangliosides (Fig. 5a). The prominin-1–GM_1_ interaction could modulate its tertiary structure and promote bending along the tubular membrane, while its oligomerization would further stimulate or stabilize the membrane curvature [237, 239]. These issues as well as the mechanism(s) regulating the dimerization/oligomerization of prominin molecules should be further addressed through biophysical investigation. The presence of a leucine zipper-like motif in the first extracellular loop (EC2) of prominin-1 could be implicated in its clustering [97].

The presence or absence of the optional exon 4, which encodes only 9 residues in the EC1 domain of prominin-1, is of interest as it could influence the interaction with GM_1_ by modulating the organization and/or the position of the GM_1_-binding site relative to the plasma membrane (Fig. 5a, b). In photoreceptor cells, the inclusion of this small exon as well as the exclusion of an IC3 domain-associated exon appear to be important for the proper function and maintenance of the retina [419–421]. Further investigation is required to determine whether the presence or absence of specific exons influence the biochemical and/or mechanical activities of prominin-1 on membrane protrusions and/or its crosstalk with cytoplasmic protein partners [78, 82, 98, 419]. It is tempting to speculate that particular splice variants of prominin-1 may have certain structural properties specific for a given type of protrusion, and may regulate the release of prominosomes.

As prominin-1 is a glycoprotein, it is worth noting that all asparagine residues found in the consensus N-glycosylation site [422], which can potentially carry N-glycans [97, 423], are on the convex side of human prominin-1 (Fig. 5c), suggesting that a differential glycosylation could potentially influence its tertiary structure [101]. Additionally, it could promote the binding of prominin-1 to other glycoproteins or glycolipid interactors, among other features [235]. The contribution of complex N-glycans through the ubiquitination of prominin-1 has been shown to affect its subcellular distribution and secretion through exosomes [424]. It is widely documented that the glycosylation profile of prominin-1 depends on the tissue and on the cellular state [78, 85, 172, 173, 425] (reviewed in Ref [56]). Thus, a given splice variant of prominin-1 could potentially organize the structure and dynamics of biological membranes through its glycosylation or other post-translational modifications.

The peculiar banana-like shape of prominin-1 extends to other members of the prominin family [58, 137, 426], such as mammalian prominin-2 (Fig. 5d) and its homologues in fruit fly and worm (Fig. 5d). In the case of invertebrate species, the bending of prominin molecules is even more evident. Interestingly, the *Drosophila melanogaster* prominin-like protein harbors a potential extra-transmembrane segment at its N-terminus [134], which would further promote its bending. This banana-like shape is somehow reminiscent of cytoplasmic proteins with Bin/amphiphysin/Rvs (BAR) and FER/Cip4 homology-BAR domains that drive the formation of positive membrane curvature as those observed in plasma membrane invaginations (reviewed in Refs [427, 428]). Interestingly, inverse (I)-BAR proteins, such as IRSp53 [429], MIM and insulin receptor tyrosine kinase substrate (IRTKS), have been reported to induce clusters of PIP_2_ lipids at the inner membrane leaflet and promote a negative membrane curvature as those found inside of filopodia, microvilli, and cilia [430–434]. It is perhaps not coincidental that IRSp53 induces, similar to prominin-1, small and transient filopodia without actin filaments [435]. Furthermore, IRSp53 and IRTKS have been shown to be released in association with ectosomes in a process dependent on the Arp2/3 complex, but independent of TSG101 [436]. Similarly, the MIM protein is associated with the release of large filopodia-derived ectosomes that can stimulate recipient cell migration via lamellipodia formation [286].

It would be interesting to assess whether prominin-1 could physically provide a scaffolding mechanism along tubular structures, as has been proposed for BAR domain proteins on positive membrane curvature, and whether the GM_1_-dependent interaction of prominin-1 with PI3K could alter PIP_2_–protein interactions on negative membrane curvature [437]. Thus, prominin-1 could act as a physical and biochemical regulator in the construction of strongly curved plasma membranes and influence the release of specific EVs, the prominosomes.

## Biological function(s) of prominosomes

Why do stem cells as well as terminally differentiated cells release prominosomes? Two distinct, but not mutually exclusive, scenarios are possible. First, together with intracellular mechanisms regulating the degradation of cellular constituents, the release of prominosomes may represent an alternative way of discarding components in specific cellular states under physiological and pathological conditions. Second, prominosomes may play a role in intercellular communication, notably over long distances. In such context, the association of prominin-1 with specific cholesterol-rich membrane microdomains and/or its involvement with various signaling pathways through its interaction with various protein and lipid partners may find some rationality. Of note, these implications do not rule out a role of other membrane cholesterol-enriched EVs lacking prominin-1 in various cellular processes. Here, we will highlight the issues, where prominosomes can be implicated.

*Cell fate determinants* – In two distinct cellular systems, i.e. neural and hematopoietic lineages, prominin-1 was initially demonstrated to be released into the extracellular milieu in association with EVs. Remarkably, this was associated with the stem cell differentiation process. In neural progenitors, the release of prominosomes could result from a significant remodeling of the apical membrane, notably of microvilli and primary cilia, and from the release of the midbody [54, 57]. Thereby, it could contribute to the reduction of the apical surface area that coincides with the transition from proliferative to neurogenic divisions [196], and hence, regulate the balance between proliferation and differentiation during development (reviewed in Ref [55]). The total amount of prominosomes released into CSF of the neural tube lumen rose at early stages of cortical development (embryonic day (E) 10.5 to E12.5) and declined thereafter (E12.5-E13.5) [54]. This fluctuation reflects respectively an increase and decrease in small prominin-1^+^ ectosomes, while the number of large ones (i.e. secreted midbody remnants) remained largely constant at the early stage and only decreased later as neurogenesis progressed [54, 57, 350]. The release of small prominin-1^+^ ectosomes from primary cilia could further contribute to the clearance of signaling molecules, and thus terminate a particular signaling pathway, and/or contribute to ciliary dynamics [57, 140, 296–298, 438, 439].

In human CD34^+^ HSPC populations, prominin-1 marks cells with more primitive stem cell properties than prominin-1^neg^/CD34^pos^ [440, 441] and the release of prominin-1^+^ exosomes has been shown to parallel their differentiation [181]. Interestingly, these exosomes did not contain CD34 [181], which is routinely used as surface marker of hematopoietic and endothelial progenitors [442] (reviewed in Ref [443]). Beyond stemness, the loss of prominin-1 could affect the morphological characteristics and/or biophysical properties of stem/progenitor cells as illustrated by the formation of narrow tunneling nanotubes between prominin-1^pos^/CD34^pos^, but not prominin-1^neg^/CD34^pos^ hematopoietic progenitors [444].

In line with data obtained from healthy cells, an in vitro study using Caco-2 cells has shown that the release of prominosomes was linked to their differentiation [54]. These findings, together with the association of prominin-1 with cholesterol-rich membrane microdomains within cells and EVs derived therefrom [83, 234], have led to the concept of “stem and cancer stem cell–specific lipid rafts” that harbor molecular components determining the stem cell state [54, 181, 234, 241]. Thus, their loss (or release) is part of, or even promoting, the cell differentiation process. In agreement with this hypothesis, it was reported that sodium butyrate stimulated colon cancer cell differentiation as well as the downregulation of the cellular expression of prominin-1 [445]. Moreover, blocking the maturation of MVBs with ammonium chloride prevented both sodium butyrate-induced differentiation and the reduction of prominin-1 expression confirming that the release of prominin-1^+^ exosomes is required for their differentiation [446].

Finally, it cannot be ruled out that the shedding of prominin-1^+^ ectosomes reflects the general turnover of cellular, notably microvillar, membranes, which could be important for the homeostasis of terminally differentiated epithelia, e.g., in the kidney and gastrointestinal tract. Membrane renewal is also illustrated for photoreceptor cells, where membrane discs are continually renewed by the growth of new prominin-1-containing membrane evaginations at the base of the outer segment. Therefore, prominin-1 expression and prominosome release could both be indicators of plasma membrane activities that influence the morphological and physiological state of prominin-1^+^ cells.

*Intercellular communication –* In general, EVs play an important role in intercellular communication and it is very likely that this is also true for prominosomes. The uptake of small prominosomes has been shown in healthy cells such as mesenchymal stromal cells or fibroblasts and in various cancer cells [181, 241, 446, 447]. Thereby, prominin-1 could influence various signaling pathways not only of donor cells, but recipient cells as well [54].

Numerous studies have documented the contribution of prominosomes to cancer progression through the promotion of tumor angiogenesis [448] or metastasis by stimulating cancer cell migration and/or transforming the cancer cell microenvironment [241, 390, 446, 449]. For example, the incubation of mesenchymal stromal cells with melanoma cell-derived small prominosomes increased their invasive capacity [241]. Similarly, human colorectal adenocarcinoma HT29 cells release prominosomes which, when taken up by rodent fibroblasts or human colon cancer cells, increase their proliferation and the motility of the latter [446]. Upon prominosomes exposure, two small GTPases were expressed, namely Rac1 and particularly Cdc42, which are crucial for inducing the formation of membrane ruffles and filopodia, respectively [446]. Their increased expression leads to the reorganization of cytoskeleton dynamics, and thereby affects motility and cell invasion [450]. After the uptake and potential endosomal recycling, prominosomes-derived prominin-1 could stimulate the migration of recipient cells through the formation of lamellipodia via its interaction with the Arp2/3 complex and PI3K. Moreover, through the production of PIP_3_, it could provoke oncogenic signaling, since PIP_3_ acts as a lipid second messenger and regulates the Akt signaling pathways among others [451]. In addition, cells treated with prominosomes show increased phosphorylation of Src and ERK proteins [446], which are known to regulate cell proliferation, EMT and, hence, adhesion and migration [452, 453]. These observations are in agreement with the implication of prominin-1 in signaling pathways involving phosphorylated Src and ERK proteins [89, 454–456] and in biological processes such as cancer cell migration and metastasis [390, 457].

Intercellular transfer of prominosomes has been shown to trigger drug resistance in non-tumorigenic recipient cells. Kang and colleagues have reported that Kirsten rat sarcoma virus oncogene homologue (KRAS)-mutated colon cancer cells can release prominosomes that activate KRAS downstream signaling in recipient cells after their uptake [449]. Thereby, prominosomes can stimulate cell migration and invasiveness, and induce the development of chemoresistance by abolishing the inhibitory actions of anti-epidermal growth factor (EGF) receptor drugs [449]. The small GTPase KRAS is one of the most frequently mutated oncogenes in human cancer that acts as an EGF receptor signaling transducer [458, 459]. Interestingly, the same study observed a connection between the expression level of prominin-1, which is stimulated by EGF signaling, and the amount of budding EVs. The relative size of EVs was also influenced by prominin-1. These indicate that prominin-1 itself can act as a modulator in the release of prominosomes (in this case ectosomes) [449]. Although the exact mechanism behind these events has not yet been elucidated, a potential link has been established with RhoA. Its activity was increased in prominin-1^+^ compared to negative cells. In contrast, the activity of another small GTPase, Rac1, was reduced [449]. This interplay between the activities of RhoA and Rac1 had already been reported, with the former promoting the shedding of EVs from the plasma membrane and the latter stimulating the formation of invadopodia, and thus participating in one way or another in the mode of migration, i.e. amoeboid or mesenchymal motility [460]. It was shown that the RhoA-ROCK/ERK signaling pathways and the contractility of actomyosin through the regulation of the myosin light chain phosphatase are involved in this cancer-specific shedding of EVs [460]. The activation of RhoA was either promoted by the inhibition of Rac1 or the activation of ADP-ribosylation factor 6 (ARF6) [449, 460]. Thus, prominin-1 could somehow be involved in these pathways to regulate EV budding. As the authors point out, prominin-1 could be considered as a therapeutic target for controlling cancer growth and metastasis in anti-EGF receptor drug-resistant colon cancer [449].

Of note, the small GTP-binding protein ARF6 was reported to stimulate the shedding of large non-apoptotic ectosomes (300–900 nm in diameter) in human melanoma cells. The shedding is facilitated through a phospholipase D and ERK axis and an actomyosin-based membrane abscission mechanism. These large ectosomes contain functional proteases and are thus involved in the degradation of the extracellular matrix [461]. Moreover, the regulation of glioma cell invasion has been associated with ARF6, which can form a complex with Rac1 and IQGAP1 [462] suggesting a potential link between prominin-1, ARF6 and their interactors in distinct but interconnected processes such as cell migration and EV release. It might be instructive to assess (i) whether ARF6 and phospholipase D are involved in the shedding of prominosomes and (ii) if the latter contain proteases involved in the degradation of the extracellular matrix. Interestingly, a protease named Adam10 (a disintegrin and metalloproteinase domain 10) that acts as a sheddase has been detected in melanoma-derived small prominosomes by a proteome analysis [241].

In the immune system, tumor-derived prominosomes could contribute to cancer progression by mediating the crosstalk between cancer cells and infiltrating macrophages leading to their polarization and transformation into M2-like tumor-associated macrophages [463]. By that, an immunosuppressive tumor microenvironment could be created that contributes to tumor growth, metastasis, tumor angiogenesis, immune suppression, and drug resistance, among other things [448, 463]. Prominosomes-primed M2-like tumor-associated macrophages showed increased secretion of cytokines and chemokines, notably interleukin-6, compared to untreated cells leading to the activation of the STAT3 pathway in EV-donor cancer cells [448]. Since interleukin-6/STAT3 signaling is known to stimulate prominin-1 expression [464], prominosome-mediated cancer cell–macrophage communication could create a positive feedback loop that further enhances cancer growth and metastasis [465]. The relationship between prominin-1, interleukin-6 and the STAT3 pathway has been intensively discussed in recent reviews [95, 96].

Besides protein cargoes, the impact of prominosomes could depend on enclosed microRNAs, which can control gene expression by base pairing with messenger RNAs [466]. For example, the miRNAs miR-10a, -24 and − 27a have been shown to be enriched in cancer cell-derived small prominosomes [241, 446] and are known for their effect on EMT and immune tolerance [467, 468] (reviewed in Ref [469]). In addition, other EV components, such as messenger or long non-coding RNAs, membrane lipids, or lipid droplets/mitochondria could contribute to the crosstalk between cells and their microenvironment, either close to the donor cell itself or over long distances, as observed in metastases [470].

The long-range activity of prominosomes can occur in non-mammalian cells and invertebrates. This has been elegantly demonstrated in *Drosophila*, where the prominin-like protein affects the EV donor membrane, i.e. the microvilli, and contributes to the proper development of the wing imaginal discs via EV-mediated long-distance Hedgehog signaling [471]. This indicates that basic biological processes that are based on prominin molecules are conserved across species as also shown for the visual system highlighted above.

*Dual role of prominosomes –* The association of prominin-1 with the midbody and its secreted form could be a good example of a case where two functions that are not mutually exclusive occur at the same time [57]. First at all, asymmetrical or symmetrical abscission along the midbody can impact the inheritance and accumulation of given factors, thereby affecting the fate of the daughter cells in different ways (Fig. 3b). In addition to their shedding, i.e. the disposal of putative determinants of stem cell fate, the secreted midbody remnant can be reabsorbed by cells via an actin-dependent phagocytosis mechanism, either by a daughter cell after cell division or by non-daughter cells in the close vicinity or at greater distance (Fig. 3b, see legend) [167, 349, 355]. In the nervous system, the uptake of secreted midbody remnants can contribute to intercellular signaling specifying cell polarity and determining cell fate [54, 57, 472, 473]. In addition, midbody and its secreted form can serve as sources for other small and large prominosomes, which further participate in intercellular communication [57, 349] (reviewed in Ref [474]).

*Are prominosomes specific entities? –* Small and large prominosomes may represent a special class of EVs, especially when prominin-1 is involved in their formation, the incorporation of selective cargoes, and/or their cellular uptake. The last two aspects are poorly documented, but prominin-1 could contribute to the selective binding and uptake of prominosomes by recipient cells, either by interacting with other prominin molecules or another molecular player. Cholesterol-dependent membrane microdomains associated with prominin-1 might be involved in the EV uptake mechanism [242]. Furthermore, the banana-shaped structure proposed for prominin molecules looks like a small hook. Reminiscent of a “velcro-like” system, its structure could facilitate numerous weak bindings that eventually result in a firm and specific interaction between prominosomes and cells. On top of that, the localization of prominins in plasma membrane protrusions has made these structures ideal targets for the contact and binding of EVs, as documented for the interaction of EVs with primary cilia or filopodia [295, 475]. Further research should define the potential adhesive role of prominins in this process, such as trans-interactions between opposing membranes. In the light of the previous sections on mouse multiciliated ependymal cells and *Drosophila* rhabdomeric microvilli, this role would appear to contradict the observed anti-adhesive functions of prominins in these systems. This discrepancy could be explained by the facultative involvement of a tissue-specific interactor (e.g., Spacemaker) that could counteract the function of prominin molecules.

From a phenotypic point of view, the selective release of prominosomes during plasma membrane remodeling could be indicative of the cellular state. The reduction or increase of prominosomes in a given body fluid could be used to monitor physiological conditions, particularly embryonic development and regeneration after injury, and pathological conditions (see next section). Therefore, it may be useful to add prominin-1 (or -2) as a classical marker when analyzing EVs, like tetraspanin membrane proteins (CD9, CD63, CD81) [161, 476]. From a functional point of view, the association of prominin-1 with cholesterol-dependent membrane microdomains and specific signaling pathways define prominosomes as specialized carriers whose release and uptake can lead to biochemical and morphological responses in donor and recipient cells [96].

Further studies are needed to decipher all properties of prominin-1^+^ EVs compared to prominin-1^−^ EVs, including their content, their stability in a given body fluid, the mechanisms of their release and/or uptake, and functional effect(s) in recipient cells. Yet, we could envision applications in tissue engineering or cancer therapy arising from the inhibition or stimulation the clearance of prominosomes, as the former could enhance stem/progenitor cell proliferation, while the latter could promote cancer cell differentiation.

## Clinical relevance of prominosomes

Given the advanced technologies now available, particularly the multi-omics approach, the search for predictive biomarkers associated with body fluids is highly relevant. Liquid biopsy analysis can provide essential information for the diagnosis and prognosis of some of the most common solid tumors [477]. Regardless of the function(s) attributed to prominosomes, their detection in CSF, urine, seminal fluid, saliva, and blood may be used as a non-invasive biomarker that could mirror the composition of donor cells and their activities, notably membrane dynamics [54]. Given that prominin-1 is upregulated in various cancers, monitoring the amount of prominosomes in a given body fluid can be highly informative and they may be used as a potential cancer biomarker [72].

In brain cancers, prominin-1 has been shown to highlight cancer stem cells [60, 61, 478–480] and prominin-1^+^ glioma cells have been shown to be resistant to radiotherapy [481]. Moreover, a high prominin-1 expression level was associated with a worse prognosis in glioblastoma patients [482]. Interestingly, Huttner and colleagues have shown an increase in small prominosome concentration in the CSF of glioblastoma patients at an early stage, followed by a dramatic decrease at the last stage of the disease [171]. This could reflect the presence of proliferative prominin-1^+^ cancer cells or an upregulation of prominin-1 in normal neural stem cells in the vicinity of the tumor at early stages followed by a differentiation of prominin-1^+^ cancer cells leading to the loss of prominin-1, and consequently a decrease in small prominosomes [171]. Thus, quantifying CSF-associated prominosomes could be useful for monitoring not only physiological brain aging, but also cancer progression [171].

Besides brain cancer, CSF is frequently used for diagnosing a variety of neurological diseases. The basic analysis of CSF includes cell count, total protein amount and biochemical and cytological findings [483, 484]. In addition, it could be of interest to assess the number of EVs in general [485], and those containing prominin-1 in particular. Along this line, patients with partial temporal lobe epilepsy [486], normal pressure hydrocephalus, parkinsonism as well as relapsing-remitting and secondary progressive multiple sclerosis [487] were found to have an increase in number of small prominosomes in the CSF. This was also the case for patients with subarachnoid and intracerebral hemorrhage, which showed a decrease in CSF-associated prominosomes during the first seven days after admission to hospital [488].

In these neural areas of biological activity, CSF-associated prominosomes could originate from ciliary structures of ependymal cells and/or underlying subventricular astrocytes that extend their primary cilium into the lumen and act as stem cells [75, 171, 489–493]. Prominin-1 is also associated with the myelin sheath formed by oligodendrocytes and Schwann cells found in the central and peripheral nervous system, respectively [82, 494]. In these glial cells, prominin-1 may play a role in myelination and, in certain diseases, the breakdown of myelin (and/or remyelination process) could contribute to the shedding of small particles containing prominin-1 [495–499]. For a general overview of prominin-1 expression in neural tissue, we refer to the following review article [500]. It remains to be clarified whether the inflammatory process itself stimulates its expression and/or the release of prominosomes. A link between prominin-1 expression and inflammation has been observed in solid cancers [170, 501] (reviewed in Ref [393]). However, prominin-1 is expressed by endothelial progenitors [502] and, if neovasculogenesis occurs during cancer progression [503], those cells may contribute to the release of prominosomes. As demonstrated for human endothelial colony-forming cells, prominosomes could additionally be of exosomal origin, as an intracellular pool of prominin-1 was observed [504]. More investigations are needed to explain the increase in number of prominosomes in the diseases mentioned above, but monitoring their presence in the CSF might be of diagnostic and prognostic interest.

In the blood, prominosomes have been postulated to be a prognostic biomarker for patients with metastatic colorectal cancer [505]. Similarly, the glycosylation state of prominin-1 associated with EVs might be a marker for pancreatic cancer diagnosis and prognosis. In detail, prominin-1 is expressed in the adult pancreas, i.e. in a subset of differentiated ductal epithelia and centroacinar cells [81, 173, 174], and in pancreatic cancer stem cells [506]. In patients with advanced pancreatic cancer, highly glycosylated forms of prominin-1 were found to be more strongly expressed in EVs collected from malignant ascites compared to those derived from non-malignant ascites from liver cirrhosis or patients with gastric cancer [507]. The authors reported preliminary data in favor of a correlation between an increase in prominin-1 glycosylation and enhanced survival of patients. They further proposed that this information could help to select patients with a better prognosis for chemotherapy as part of an advanced pancreatic cancer treatment regime [507].

Furthermore, prominosomes might be used for monitoring renal failure and/or functional recovery upon tissue engineering and/or cell replacement. This is especially relevant as EVs can in some way reflect the condition and function of the tissue from which they originate. This is well illustrated by the exciting research of Bussolati’s group and others, showing that prominin-1 associated with urinary EVs could be used to monitor acute and chronic glomerular injuries, among other kidney diseases [508, 509] or tissue recovery after kidney transplantation [510, 511] (reviewed in Ref [512]). In the kidney, prominin-1 is associated with cells found in proximal tubules and the parietal layer of Bowman’s capsule of juxtamedullary nephrons [58, 172]. It highlights cells with stem cell properties [67, 513–516], and perhaps, those with dedifferentiation capacities that act as facultative stem cells [517, 518]. Urinary EVs that contain prominin-2 may also have diagnostic and prognostic utility [519]. In contrast to prominin-1, prominin-2 is associated with distal tubules [158]. Hence, both EV-associated prominin molecules can be exploited to monitor the activity of distinct regions of the nephron. The same rationale can be applied to saliva-derived prominin-1^+^/-2^+^ EVs to monitor salivary gland stem cell transplantation or cancer [155, 170, 177, 520].

In addition to their potential use as biomarkers, prominosomes may be used as bioactive agents to improve health conditions, e.g., in cardiovascular diseases, as has been suggested for EVs originating from mesenchymal stromal cells or cardiac progenitors [521–526]. Indeed, various preclinical stroke models have underlined the neuroprotective effects of EVs derived from various cellular sources (reviewed in Ref [527]). For example, Venkat and colleagues have demonstrated that the intravenous injection of EVs derived from prominin-1^+^ human umbilical cord blood cells into mice with type 2 diabetes mellitus that were subjected to stroke significantly improved neurocognitive outcomes and reduced cardiac and hepatic dysfunction. This is especially important considering the higher incidence of stroke in patients with diabetes mellitus and the adverse effects of this condition on the function of other peripheral organs. It further suggests that these EVs could find application as a therapeutic agent in regenerative medicine [528, 529]. This is a pertinent consideration, as cell-free based therapies avoid any complications associated with the use of living cells [530]. Moreover, the transfer of certain cargoes (e.g., miR-126) mediated by prominosomes could stimulate the angiogenic response and increase the capillary density, as well as affect the expression of inflammatory factors [528]. These findings are consistent with potential myocardial regeneration through vascular endothelial growth factor that is enhanced after bone marrow multipotent prominin-1^+^ stem cell therapy [531–533]. However, it was shown that tail vein-injected prominosomes derived from isolated prominin-1^+^ renal progenitors did not contribute to kidney repair in a mouse model after ischemia-induced acute kidney injury, whereas isolated prominin-1^+^ cells themselves or EVs derived from glomerulus-associated mesenchymal stromal cells could, with the latter mimicking the effect of their cell of origin [534].

In all clinical cases, the use of prominin-1^+^ (or perhaps prominin-2^+^) EVs associated with a given body fluid as biomarkers will require further evaluation in a large cohort of patients. Their role as potential bioactive agents will merit further investigations in animal models, including the use of prominin-1-deficient animals and murine prominosomes.

For alternative applications, prominin-1 itself could serve as a molecular target either for immunotherapies or to enable the directed delivery of drug-containing liposomes. Furthermore, prominosomes could be exploited as specific vehicles for drug delivery [535–539] as they can potentially be internalized by specific target cells that differ to the ones that take up prominin-1^−^ EVs. The interception of prominosomes-mediated intercellular crosstalk, particularly in cancer, where EV-associated prominin-1 and other cargoes can induce proliferation, EMT and cancer cell invasion, could be of interest. One approach could be the use of blocking antibodies that neutralize the uptake of these prominosomes, as has been suggested for another EV-associated, the tetraspanin protein CD9 [540, 541]. Of note, all potential applications of prominosomes described in this paragraph are not mutually exclusive.

## Summary and prospect

Since its discovery in 1997, prominin-1 has revealed new properties of stem cells and terminally differentiated epithelial cells, in particular the release of small and large ectosomes from various types of membrane protrusions such as microvilli, primary cilia, midbodies and cell extremities. Its release in association with exosomes has been correlated with the differentiation of HSPCs. It remains to be investigated whether other cell types that express prominin-1, including oligodendrocytes, astrocytes, and spermatozoa, also release small or large prominosomes [542–544]. Similarly, it might be interesting to re-evaluate the potential association of prominin-1 with EVs in various published studies where its expression has not been scrupulously analyzed. As prominin-1 has been found to regulate the structure of plasma membrane outgrowths, their dynamics, and perhaps their composition, the analysis of prominosomes in a given body fluid might be instructive about the physiological activity of the donor cells. The effect of prominosomes on receiving cells and/or their involvement in certain pathologies could eventually reveal new cellular characteristics mediated by their intercellular transfer. Monitoring the uptake of prominosomes and the fate of their cargoes will be of interest as the various types of EVs might regulate specific pathways.

In the case of cancer, prominosomes have been demonstrated to favor cancer growth and metastasis, and it may therefore be of medical interest to find ways of selectively preventing their release and/or uptake. In addition to prominin-1, other members of the prominin family, across species, deserve particular attention, as they may extend the characteristics of prominin-1 to various other tissues and organs. This is particularly true for prominin-2, which is associated with the basolateral domain of epithelial cells and is released in association with small prominosomes. Collectively, studies of prominin-1 (or other related prominin family proteins) have revealed distinct types of EVs originating from different cellular sources, which together may represent a particular class of EVs.

## Conclusion

The interactions of prominin-1 with various lipids and proteins affect highly curved membrane structures and their dynamics, mainly through the release (or non-release) of prominosomes. These interactions may implicate prominin-1 in numerous biological processes during development and adulthood that require the remodeling of the plasma membrane under healthy and diseased conditions. Increasing our knowledge about prominin-1 and prominosomes could therefore shed light on novel and/or intertwined molecular and cellular pathways. In addition, it might reveal new options for the promotion of stem cell-based tissue regeneration or the interception of disease progression, particularly in cancer.

## Electronic supplementary material

Below is the link to the electronic supplementary material.

Supplementary Material 1

## Data Availability

No datasets were generated or analysed during the current study.

## Abbreviations

Akt: Rac-alpha serine/threonine-protein kinase
Alix: ALG-2-interacting protein X
ARF6: ADP-ribosylation factor 6
Arl13b: ADP-ribosylation factor-like 13b
Arp2/3: Actin-related protein 2/3
BAR: Bin/amphiphysin/Rvs
BBS: Bardet-Biedl syndrome
CD: Cluster of differentiation
CHO: Chinese hamster ovary
CRAC: Cholesterol recognition/interaction amino acid consensus
CSF: Cerebrospinal fluid
EC: Extracellular
EGF: Epidermal growth factor
EMT: Epithelial-mesenchymal transition
ERK: Extracellular signal-regulated kinase
ERM: Ezrin/radixin/moesin
ESCRT: Endosomal sorting complex required for transport
EVs: Extracellular membrane vesicles
GLIS2: GLI-similar 2
GM_1_: Monosialotetrahexosylganglioside
GM_3_: Monosialodihexosylganglioside
GPI: Glycosylphosphatidylinositol
HDAC6: Histone deacetylase 6
HSPCs: Hematopoietic stem and progenitor cells‹
IC: Intracellular
ILVs: Intralumenal vesicles
IQGAP1: IQ motif containing GTPase-activating protein 1
IRSp53: Insulin receptor substrate p53
IRTKS: Insulin receptor tyrosine kinase substrate
KRAS: Kirsten rat sarcoma virus oncogene homologue
mβCD: Methyl-β-cyclodextrin
MDCK: Madin-Darby canine kidney
MIM: Missing-in-metastasis
MVBs: Multivesicular bodies
NE: Neuroepithelial
NPHP7: Nephrocystin-7
PDZ: PSD95, DIgA, Zo-1
PI3K: Phosphoinositide 3-kinase
PIP_2_: Phosphatidylinositol 4,5-bisphosphate
PIP_3_: Phosphatidylinositol 3,4,5-trisphosphate
PLAP: Placental alkaline phosphatase
ROCK: Rho-associated coiled-coil containing protein kinase
SEM: Scanning electron microscopy
STAT3: Signal transducer and activator of transcription 3
TGN: Trans-Golgi network
TM: Transmembrane
TSG101: Tumor susceptibility gene 101
WAVE: Wiskott-Aldrich syndrome protein family verprolin-homologous protein
WGA: Wheat germ agglutinin
WT: Wild-type
