# Supplementary figures and images for: Prominosomes - a particular class of extracellular vesicles containing prominin-1/CD133?
Source: J Nanobiotechnology. 2025 Jan 29;23:61. doi: 10.1186/s12951-025-03102-w (PMC11776279; doi:10.1186/s12951-025-03102-w)

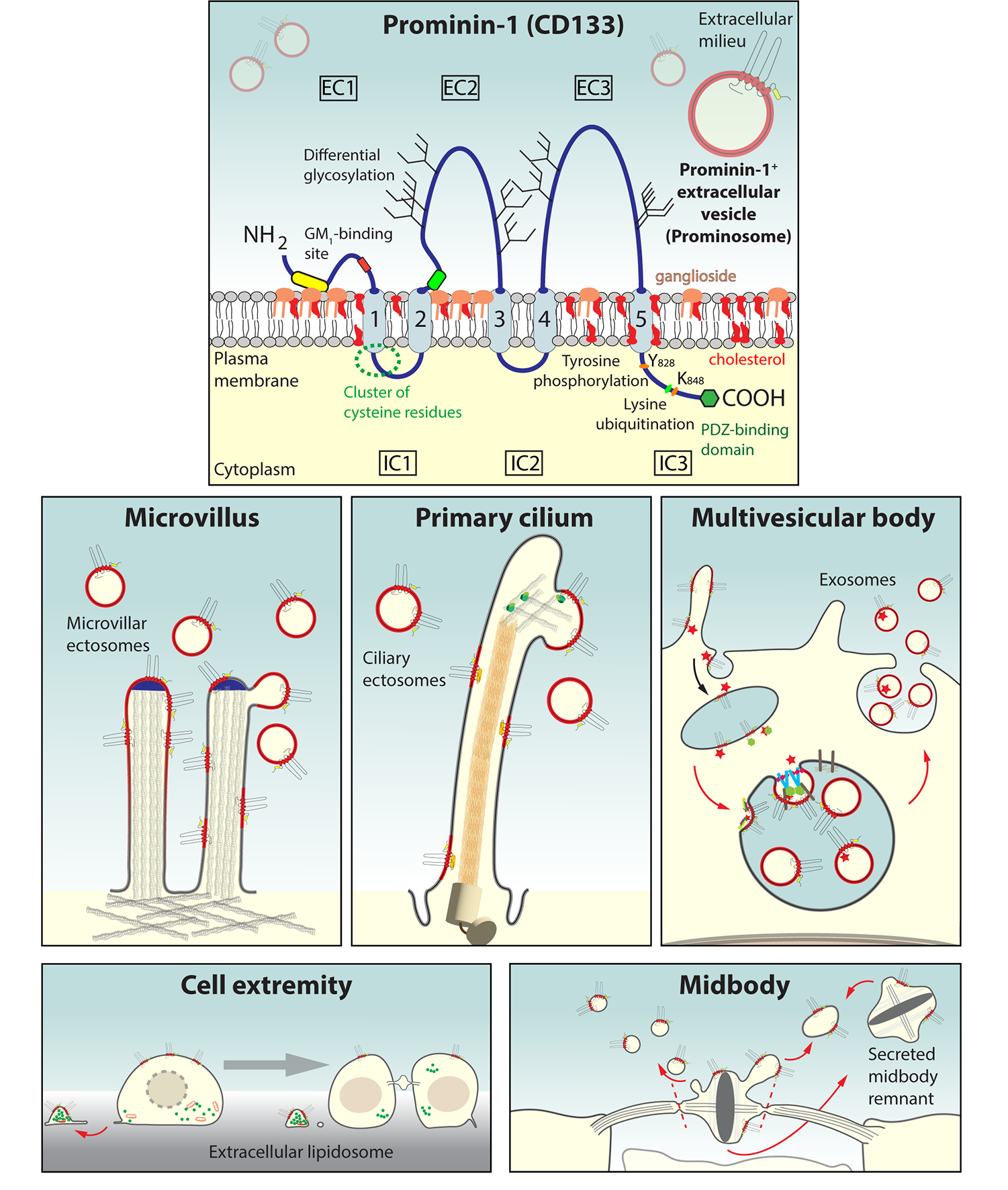

Supplement: Supplementary file 1 — Supplementary Material 1 [file 12951_2025_3102_MOESM1_ESM.tif]
